# Culture-Dependent and -Independent Analyses Reveal the Diversity, Structure, and Assembly Mechanism of Benthic Bacterial Community in the Ross Sea, Antarctica

**DOI:** 10.3389/fmicb.2019.02523

**Published:** 2019-11-08

**Authors:** An-Zhang Li, Xi-Bin Han, Ming-Xia Zhang, Yang Zhou, Meng Chen, Qing Yao, Hong-Hui Zhu

**Affiliations:** ^1^State Key Laboratory of Applied Microbiology Southern China, Guangdong Provincial Key Laboratory of Microbial Culture Collection and Application, Guangdong Microbial Culture Collection Center, Guangdong Institute of Microbiology, Guangdong Academy of Sciences, Guangzhou, China; ^2^Key Laboratory of Submarine Geosciences, Second Institute of Oceanography, Ministry of Natural Resources, Hangzhou, China; ^3^Guangdong Province Key Laboratory of Microbial Signals and Disease Control, Guangdong Engineering Research Center for Grass Science, Guangdong Engineering Center for Litchi, College of Horticulture, South China Agricultural University, Guangzhou, China

**Keywords:** Ross Sea, bacterial diversity, community structure, microflora, community assembly, high-throughput sequencing, Antarctica, assembly mechanism

## Abstract

The benthic bacterial community in Antarctic continental shelf ecosystems are not well-documented. We collected 13 surface sediments from the Ross Sea, a biological hotspot in high-latitude maritime Antarctica undergoing rapid climate change and possible microflora shift, and aimed to study the diversity, structure and assembly mechanism of benthic bacterial community using both culture-dependent and -independent approaches. High-throughput sequencing of 16S rRNA gene amplicons revealed 370 OTUs distributed in 21 phyla and 284 genera. The bacterial community was dominated by *Bacteroidetes, Gamma*- and *Alphaproteobacteria*, and constituted by a compact, conserved and positively-correlated group of anaerobes and other competitive aerobic chemoheterotrophs. Null-model test based on βNTI and RCBray indicated that stochastic processes, including dispersal limitation and undominated fractions, were the main forces driving community assembly. On the other hand, environmental factors, mainly temperature, organic matter and chlorophyll, were significantly correlated with bacterial richness, diversity and community structure. Moreover, metabolic and physiological features of the prokaryotic taxa were mapped to evaluate the adaptive mechanisms and functional composition of the benthic bacterial community. Our study is helpful to understand the structural and functional aspects, as well as the ecological and biogeochemical role of the benthic bacterial community in the Ross Sea.

## Introduction

Marine sediments represent the largest reservoir of organic carbon and consequently support abundant and diverse benthic bacterial communities (Kallmeyer et al., 2012), which play fundamental roles in food web structure, energy flow and global biogeochemical cycle (Sunagawa et al., 2015). Particularly in extreme environments, such as Antarctic continental shelves, the role of bacteria appears to be even more important because they are considered as the predominant or even sole components and dominate the biomass and taxonomic diversity in Antarctic ecosystems (Wynnwilliams, 1996; Convey, 2001).

So far, only few studies described the diversity and community composition of bacteria in Antarctic benthic ecosystems, partly due to the limitation of sampling (Sunagawa et al., 2015). Previously, the benthic bacterial diversity of shallow sediments collected around Australia's Casey Station (66°17′S, 110°32′E) at 10–30 m depth (Powell et al., 2012) and along the Terra Nova Bay (74°37′-74°46′S, 163°59′-164°07′E) at 20–48 m depth (Baldi et al., 2010) were studied by constructing 16S rRNA gene libraries. Recently, high-throughput sequencing based on 16S rRNA gene amplicons has been widely applied to microbial ecology. Franco et al. found that 15 marine sediments collected from Admiralty Bay (62°5′S−62°13S, 58°22′W−58°29′W, depth 100–502 m) and North Bransfield Basin (62°10′S−62°18S, 58°10′W−58°19′W, depth 693–1,147 m) near King George Island had a high abundance of *Gammaproteobacteria* (92.4% in the bay and 83.8% in the basin) (Franco et al., 2017). Carr et al. revealed that *Bacteroidetes, Gamma-* and *Alphaproteobacteria* dominated the bacteria assemblages in a sediment column collected from offshore area adjacent to the French station Dumont d'Urville (66°40′ S, 140°1′ E) (Carr et al., 2015). Studies of benthic bacterial communities in higher latitude maritime areas of Antarctica mainly focused on the Ross Sea, yet only few individual sediments were studied. Carr et al. studied the microbial abundance, diversity and community composition of a sediment core collected from a location beneath the Ross Ice Shelf (ice thickness 82 m, water depth 850 m), and found that *Beta-, Delta-*, and *Gammaproteobacteria, Actinobacteria, Chloroflexi*, and *Bacteroidetes* showed high abundance (Carr et al., 2013). Learman et al. revealed that the benthic microbial communities of Western Antarctica (including three stations in the Ross Sea) were dominated by *Gamma-, Delta-, Alphaproteobacteria, Bacteroidetes, Verrucomicrobia, Planctomycetes*, and *Actinobacteria* (Learman et al., 2016). In this context, more comprehensive studies of benthic bacterial communities focusing on a typical and integrated continental shelf area in high-latitude Antarctica are still requisite to understand the processes and functions of benthic ecosystem (Sunagawa et al., 2015).

Understanding the mechanism of community assembly is one of the most important objectives of ecology. Based on niche theory, several studies attempted to figure out the fateful role of environmental factors on shaping the microbial community structure in sediments of Antarctic continental shelf. Yet definitive and clear conclusions haven't been drawn. Learman et al. indicated that nutrient quantity and quality were correlated with bacterial community structure in sediments of Western Antarctica, according to NMDS ordination and correlation analysis (Learman et al., 2016). Franco et al. suggested that organic matter input was correlated with bacterial composition in surface sediments collected from Admiralty Bay and North Bransfield Basin, also based on NMDS ordination (Franco et al., 2017). However, Carr et al. found no correlation between particulate organic carbon and bacterial abundance in a sediment core beneath the Ross Ice Shelf according to correlation analysis (Carr et al., 2013). Jamieson et al. suggested that input of particulate organic matter didn't alter the composition, structure and evenness of bacterial communities in two deep-sea sediments collected near Crozet Islands, Southern Ocean (Jamieson et al., 2013). Recently, geographical distance was also suggested to regulate microbial community in marine sediments, but results are still controversial and contradictory (Zhou et al., 2016; Dong et al., 2017). Therefore, further study on the driving force and mechanism of community assembly in Antarctic continental shelf sediments is crucial.

The Ross Sea is the southernmost sea on this planet, representing a unique continental shelf ecosystem under extreme climate conditions. Although covered by pack ice for most of a year, the Ross Sea is one of the most biologically productive ecosystems in the entire Southern Ocean, supporting relatively high biological diversity (Wing et al., 2012; Smith and Kaufman, 2018). Beforetime, the Ross Sea was regarded as one of the last maritime areas on Earth which remains unaffected by human activity, pollution and alien species invasion. However, recent studies found that West Antarctica including the Ross Sea is one of the most rapidly warming regions on Earth (Bromwich et al., 2013; Hughes et al., 2017). Global climate change and rapid warming have given rise to sea-ice loss, increase of glacial/terrestrial inputs, algae/phytoplankton blooms, and subsequently changed the primary productivity, organic nutrients and microflora structure in Antarctic seas (Arneborg et al., 2012; Christner et al., 2018). Because the energy and carbon source supporting benthic ecosystems was mainly originated from surface water and transported downward, the benthic communities also possibly responded to climate changes (Barnes et al., 2018; Wing et al., 2018).

**Figure 1:**
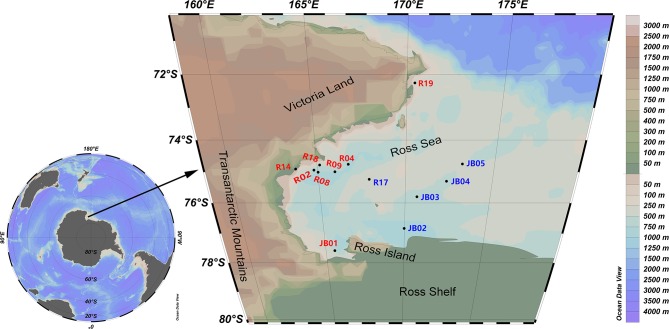
Sampling map showing the Ross Sea, Antarctica. Red, inshore stations; blue, offshore stations.

During the 32th Scientific Expedition to Antarctica of China, two groups of surface sediments were collected from the western Ross Sea (72°15′16″-77°35′18″S, 163°45′54″-173°11′17″E) (Figure 1). The inshore sampling stations expanded from the northern border of the Ross Sea near Cape Adare to the Southern border near the Ross Ice Shelf and Ross Island, along the Victoria Land. The offshore sampling stations located at the Central Trough and Coulman High. The objectives of this study are to (1) delineate the bacterial diversity and community structure in sediments of the Ross Sea using both culture-dependent and -independent approaches, (2) reveal the factors driving diversity patterns and the mechanisms underlying community assembly.

## Materials and Methods

### Sampling

Marine surface sediments were collected using box corer at 13 stations from the Ross Sea in February 2015. Each sample was divided into aliquots with a sterile trowel, and stored in sterilized plastic containers at 4 and −80°C, respectively. Salinity, temperature, pH, depth, chlorophyll and chromophoric dissolved organic matter (CDOM) were measured *in situ* using a ship-borne Seabird/Seapoint measurement system (WA, USA).

### Isolation and Purification of Heterotrophic Bacteria

Two gram of each sediment stored at 4°C was measured out with a stainless steel spoon and subsequently resuspended in 20 mL sterile seawater. The suspension were serially diluted and spread on Marine Agar 2216 (MA, BD Difco, MD, USA), MC (Li et al., 2017a,b, 2018a,b,c) and LC (1/100 MC) plates. The suspension plating process was performed in parallel to generate two analogous groups of plates, which were incubated at 4°C for 30 days and at 25°C for 7 days, respectively. Bacterial colonies were picked up from the plates and subsequently purified by repeated streaking. The 16S rRNA genes of purified strains were amplified with 27F/1492R and sequenced by Majorbio (Shanghai, China). Seawater was also diluted and spread on plates as negative controls for growth experiments following the same protocol.

### DNA Extraction, PCR Amplification, and High-Throughput Sequencing

Genomic DNA of each sample was extracted from three 0.25 g aliquots using MoBio PowerSoil DNA Isolation Kit (MoBio, CA, USA) and subsequently mix together. The V4–V5 region of 16S rRNA gene was amplified using inside primers (5′-TTCCCTACACGACGCTCTTCCGATCT-GTGCCAGCMGCCGCGGTAA-3′ and 5′-GAGTTCCTTGGCACCCGAGAATTCCA-CCGTCAATTCMTTTGAGTTT-3′). The PCR products were purified using AxyPrep™ DNA Gel Extraction kit (AXYGEN, Hangzhou, China), and subsequently amplified using outside primers to add sequencing primers, barcodes and adapters. After gel-purification, the copies of target fragments were quantified using FTC-3000 Real-Time PCR system (Funglyn, Toronto, Canada). The DNA library (2 × 300 bp) was sequenced on an Illumina MiSeq platform (Illumina, CA, USA) in TinyGene (Shanghai, China).

### Sequence Processing

The raw data containing complete barcode sequences were separated into fastq files to obtain valid reads. The paired-end sequences were merged and quality filtered using the Usearch commands of fastq_mergepairs and fastq_filter (version 10.0.240), respectively (Edgar, 2010). The UPARSE-OTU algorithm was used to generate operational taxonomic units (OTUs) at a 97% identity cutoff (Edgar, 2013). An online amplicon sequence analysis pipeline (http://mem.rcees.ac.cn) was used to plot the Venn diagram, taxonomically classify the representative sequences in SILVA database version 132, and infer species functions with FAPROTAX (Louca et al., 2016; Feng et al., 2017). α-Diversity (Shannon, Chao1, ACE, Simpson indices, observed OTUs), Good's coverage and rarefaction curves were estimated using “diversity” plugin of QIIME2 (version 2018.6). The 370 representative sequences of OTUs generated from high-throughput sequencing and the 16S rRNA genes of 629 isolates were mixed together and clustered into OTUs using CD-HIT-EST with a sequence identity cutoff of 97%. An ML tree was constructed using IQTREE web server (http://iqtree.cibiv.univie.ac.at).

### Statistical Analysis

*T*-test and ANOSIM were used to test the differences for single and multiple parameters, respectively, between the inshore and offshore groups. The alpha diversity (including Shannon, Chao1, ACE, Simpson indices) were estimated using QIIME2 with rarified OTU table (Bolyen et al., 2019). Principal coordinates analysis (PCoA) and non-metric multidimensional scaling (NMDS) were performed using PAST 3.20. Analysis of composition of microbiomes (ANCOM) was implemented using QIIME2 to compare abundances of the taxa at family and genus levels between inshore and offshore groups (Mandal et al., 2015). Network analysis was conducted using Cytoscape version 3.6.1 (Shannon et al., 2003). Mantel test and Spearman's rank correlation analysis were accomplished using online pipeline (http://mem.rcees.ac.cn) based on R to determine the correlations between geographic distance, environmental variables, α- and β-diversity. Permutation test of multivariate homogeneity of group dispersions (variances) was performed using the function “betadisper” of R (version 3.5.1) package “vegan” (version 2.5.2).

A null-model-based statistical framework was applied to determine the relative impact of stochastic and deterministic processes on community assembly (Stegen et al., 2013; Zhou and Ning, 2017). β nearest-taxon index (βNTI) based on null model test of the phylogenetic β-diversity index βMNTD (β mean nearest-taxon distance), and Raup-Crick index (RC_Bray_) based on null model test of the Bray-Curtis taxonomic β-diversity, were calculated using the R package “picante” (version 1.7) and “vegan” (version 2.5-2).

## Results

### Geochemical Characteristics of the Sediments

**Figure 2:**
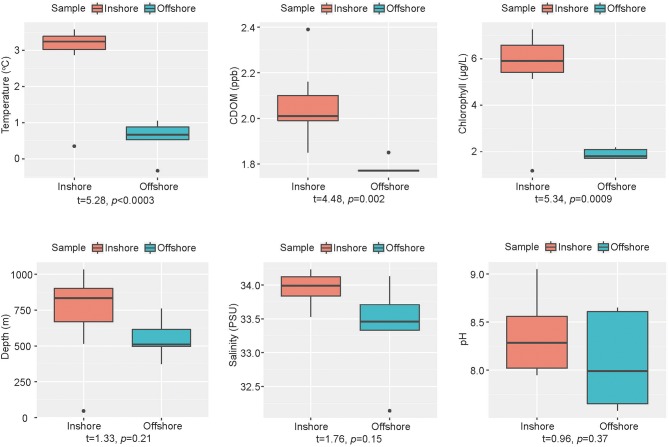
Boxplots of environmental variables between groups. Orange, inshore group; blue-green, offshore group. Welch's *t*-test between two groups was performed by R.

Geographical and chemical information of the 13 sediment samples were presented in Table S1 and Figure 2. The inshore samples (JB01, R02, R04, R08, R09, R14, R18, and R19) had significantly higher temperature (*p* < 3e-4), concentration of chlorophyll (*p* = 9e-4) and CDOM (*p* = 0.002) than the offshore samples (JB02, JB03, JB04, JB05, and R17). Whereas, salinity, pH and depth showed no significant difference between the two groups of samples. According to Spearman's rank correlation analysis (Table S2), temperature was positively correlated with the concentration of chlorophyll (ρ = 0.95, *p* < 1e-6), CDOM (ρ = 0.77, *p* = 0.002), and salinity (ρ = 0.61, *p* = 0.028). In addition, the concentration of chlorophyll was positively correlated with CDOM (ρ = 0.69, *p* = 0.009).

### α- and β-Diversity

After sequencing and quality check, 450,029 valid reads were retrieved from 13 sediment samples (mean = 34,618, ranged from 20,998 to 38,899). After paired-reads assembly, quality filtering and chimera removal, 284,963 sequences (mean = 21,920, ranged from 13,710 to 26,243), with an average length of ca. 415 bp, were finally obtained and subsequently clustered into 370 OTUs (Table S3). The inshore group and offshore group contained 366 and 175 OTUs, respectively, with an overlap of 171 OTUs. That is to say, 4 out of 175 offshore OTUs were found only in offshore samples, without parallel counterpart in inshore sediments (Figure S1).

For all 13 samples, the Good's coverage values ranged from 99.81 to 99.93% (Table S3), and the rarefaction curves were asymptotic (Figure S2), indicating that overwhelming majority of the bacteria were covered. The species richness was presented by non-parametric estimator Chao1 (ranged from 42.33 to 292.00) and ACE (ranged from 42.42 to 283.80) (Table S3). The Shannon and Simpson indices, varied from 1.91 to 5.75 and 0.53 to 0.96, respectively, were used to evaluate the species diversity. According to the α-diversity indices, the inshore samples showed higher species richness (*p* < 1e-4 based on Chao1 index; *p* < 7e-7 based on ACE index) and diversity (*p* < 5e-8 based on Shannon index; *p* < 8e-12 based on Simpson index) than the offshore samples. Specifically, the highest richness and diversity occurred at station R02, whereas the lowest were found at station R17.

**Figure 3:**
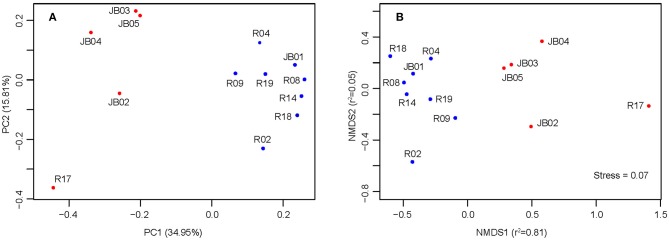
PCoA **(A)** and NMDS **(B)** analysis based on Jaccard distance. Blue, inshore sediments; red, offshore sediments.

Both PCoA and NMDS analyses suggested that inshore sediments could be clearly separated from offshore ones, mainly along the coordinate PC1 and NMDS1, respectively (Figure 3). The first and second principal coordinate of PCoA (PC1 = 34.95%, PC2 = 15.81%) explained 50.76% of the total variation. According to Figure 3, R17 was most isolated from other samples. ANOSIM analysis statistically supported the significant distinction between inshore and offshore samples (*R* = 0.51, *p* < 0.003). The results of permutational analysis of group dispersion (PERMDISP) (Anderson, 2006) revealed no significant difference in distribution divergence based on Jaccard distance between inshore and offshore groups (*t*-test = 0.47, *p* = 0.65) (Figure S3).

### Taxonomic Composition

The 370 OTUs were classified into 21 phyla, 42 classes, 107 orders, 174 families, and 284 genera. In total, *Bacteroidetes* (46.54%, ranged between 15.60 and 76.23%) and *Proteobacteria* (46.52%, ranged between 15.02 and 79.05%) dominated the overall bacterial community, followed by *Firmicutes* (2.14%, ranged between 0.07 and 11.06%), *Fusobacteria* (2.04%, ranged between 0.06 and 12.19%) and *Planctomycetes* (1.44%, ranged between 0 and 11.17%) (Figure S4). These 5 phyla (>1%) accounted for 98.67% of the total bacterial community. *Proteobacteria* (188 OTUs) and *Bacteroidetes* (78 OTUs) were also the most diverse phyla. At the class level, *Bacteroidia* (45.92%), *Gammaproteobacteria* (34.15%), *Alphaproteobacteria* (11.25%), *Fusobacteriia* (2.04%), *Clostridia* (1.78%), and *Deltaproteobacteria* (1.11%) were the most abundant classes (>1%). *Bacteroidetes, Gammaproteobacteria, Alphaproteobacteria, Firmicutes, Fusobacteria*, and *Actinobacteria* were shared in all 13 sediments.

*Bacteroidetes* was predominated by *Flavobacteriales* (33.53%, mainly *Flavobacteriaceae*), *Bacteroidales* (10.68%, mainly *Bacteroidaceae*), and *Cytophagales* (1.35%, mainly *Cyclobacteriaceae*). *Gammaproteobacteria* was mainly comprised of *Alteromonadales* (16.78%), *Oceanospirillales* (6.14%), *Pseudomonadales* (4.33%) and *Thiotrichales* (1.84%). Most *Alphaproteobacteria* were classified to *Sphingomonadales* (5.36%), *Rhodobacterales* (1.78%), and *Rhodospirillales* (1.06%). Most sequences of *Firmicutes* belonged to *Clostridiales* (1.78%, mostly *Lachnospiraceae*). All *Fusobacteria* (2.04%) sequences belonged to the genus *Fusobacterium*.

At the genus level, *Marinobacter* (13.92%), *Bacteroides* (10.37%), *Salegentibacter* (7.78%), *Bizionia* (7.70%), an uncultured *Cryomorphaceae* genus (7.45%), *Maribacter* (3.61%), *Pseudomonas* (2.60%), *Fusobacterium* (2.04%), *Escherichia-Shigella* (1.24%), and *Halomonas* (1.19%), with abundance >1%, were shared in all 13 samples. Other abundant genera included *Gillisia* (5.47%), *Sphingorhabdus* (4.07%), *Idiomarina* (2.57%), *Cobetia* (2.39%), *Arenibacter* (1.86%), *Alcanivorax* (1.74%), *Psychrobacter* (1.65%), *Sulfitobacter* (1.23%), and *Altererythrobacter* (1.13%).

Some taxa, such as *Alcanivoracaceae, Cytophagales, Cyclobacteriaceae, Caulobacterales, Magnetospiraceae, Hyphomonadaceae, Cellvibrionales, Pseudohongiellaceae*, and *Rhizobiales* were significantly more abundant in inshore sediments than in offshore ones (*p* < 0.05, *t*-test) (Figure S5). By contrast, *Flavobacteriaceae, Pseudomonadales, Moraxellaceae*, and *Psychrobacter* showed higher abundance in the offshore sediments (*p* < 0.05, *t*-test). A more rigorous methodology (ANCOM) that accounts for compositional constraints indicated that the abundance of *Microbacteriaceae* and *Psychrobacter* differed significantly between inshore and offshore groups (Table S4).

### Network Analysis and Imputed Metabolic Functions

**Figure 4:**
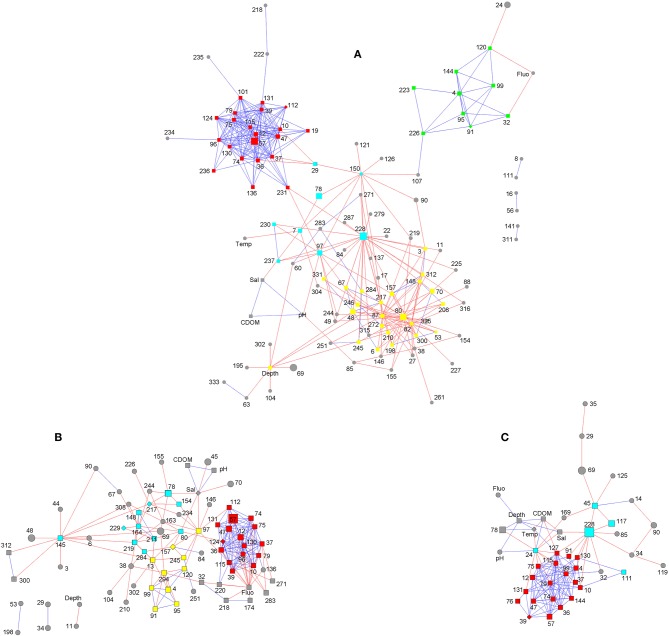
Network analysis of bacterial community in sediments of the Ross Sea. **(A)**, in all 13 sediments; **(B)**, in inshore sediments; **(C)**, in offshore sediments. Red line, negative interaction; blue line, positive interaction; square, clustered OTUs; diamond, seed OTUs; circular, unclustered OTUs; OTUs with the same color constitute a module generated by clustering algorithm.

Network analysis revealed a compact and mostly positively-correlated module, which was conserved in both inshore and offshore groups (Figure 4). The OTUs comprised in this module were annotated as *Bacteroides* (OTU12, OTU57, OTU74, OTU75, and OTU130), *Lachnospiraceae* (OTU10, OTU36, OTU39, OTU115, and OTU131), *Phascolarctobacterium* (OTU37), *Fusobacterium* (OTU47), and *Parabacteroides distasonis* (OTU79), which are anaerobic taxa according to Bergey's Manual of Systematic Bacteriology (De Vos et al., 2011; Krieg et al., 2011).

Besides this module, other OTUs mainly showed flexible competitive relationships. In inshore group, the competitive taxa were mainly connected by OTU145 (*Muricauda*), OTU97 (*Maribacter*), OTU164 (*Rhodospirillaceae*), OTU13 (*Erythrobacteraceae*), OTU294 (*Balneola*), OTU80 (*Marinobacter*), OTU217 (*Phycisphaeraceae*), OTU78 (*Gillisia*), and OTU219 (*Roseivirga*) (Figure 4B). In offshore group, the competitive taxa were less numerous, with the key nodes OTU228 (*Bizionia*), OTU24 (*Marinobacter*), and OTU45 (*Sphingomonadales*) (Figure 4C). In all 13 sediment samples of the Ross Sea, OTU80 (*Marinobacter*), OTU228 (*Bizionia*), OTU87 (*Psychrobacter pacificensis*), OTU82 (*Psychrobacter*), OTU150 (*Erythrobacteraceae*), and OTU97 (*Maribacter*) were key nodes connecting the competitive taxa (Figure 4A). These OTU nodes were the most powerful competitors in the community and were classified as aerobic chemoorganotrophs according to the SILVA database and Bergey's Manual of Systematic Bacteriology, suggesting that oxygen and organic matter were important resources in the hypoxia and low-nutrient benthos of Antarctic waters. OTUs of psychrophiles (OTU82 and 87) were not as abundant as OTU228, 57, 80, and 78, however, they were negatively correlated with 19 and 24 other OTUs, respectively (Figure 4A), indicating that psychrophiles were competitive in polar oceanic sediments.

FAPROTAX was used to map prokaryotic taxa to metabolic and ecological functions (Table S5). Most of the OTUs were assigned as chemoheterotrophs (79.49%) and aerobic chemoheterotrophs (66.43%), including most of the abundant taxa, such as *Alteromonadales, Oceanospirillales, Sphingomonadales, Flavobacteriaceae*, and *Cyclobacteriaceae*. These taxa have been reported as widespread and abundant degraders of dissolved organic carbon in seawater and marine sediments (Bowman et al., 2003; Wietz et al., 2015; Franco et al., 2017). According to function assignment, more chemoheterotrophs (97.77 vs. 67.29%, *p* < 0.007) and aerobic chemoheterotrophs (92.04 vs. 49.37%, *p* < 0.003) were predicted in offshore group than in inshore group. Moreover, bacteria associated with anaerobic fermentation accounted for 19.68% in inshore group and 14.41% in offshore group, respectively, but with no significant difference as evaluated by Welch's *t*-test. Some possible adaptive mechanisms of the bacterial community to environmental stresses were also revealed, such as nitrate/nitrite reduction, fumarate respiration, sulfur respiration, arsenate respiration, dark sulfur oxidation, manganese oxidation, utilization of xylan, cellulose, chitin, hydrocarbon, plastic, and C_1_ compounds.

### Community Assembly Mechanism

A null-model-based statistical and quantitative framework was used to disentangle the community assembly mechanism and quantify the relative importance of the stochastic and deterministic processes (Stegen et al., 2013; Zhou and Ning, 2017).

Firstly, the phylogenetic β-diversity index βNTI was tested by null model analysis (Table 1). The βNTI between JB01 and JB02 was calculated as 2.45, indicating that these two samples had greater phylogenetic turnover and more phylogenetically diverse communities, compared with null expectations. Thus, heterogeneous selection, which leads to less similar communities in phylogeny, was inferred to contribute to the community variation between JB01 and JB02. For the pairwise comparison of R17 and R19, the βNTI value of −2.13 suggested that the phylogenetic turnover was less than null expectations, and the ecological process in controlling community turnover between these two samples was homogeneous selection, which would lead to phylogenetically more similar communities. The βNTI values of all other pairwise comparisons were between −2 to 2 and not significantly different from null expectations, suggesting that the pairwise turnovers were governed by stochastic processes.

**Table 1:** Community assembly mechanisms across all pairwise combinations of communities.

**Sample**	**JB01**	**R02**	**R04**	**R08**	**R09**	**R14**	**R18**	**R19**	**JB02**	**JB03**	**JB04**	**JB05**	**R17**
JB01		1.00	1.00	1.00	1.00	1.00	1.00	0.99	1.00	1.00	1.00	1.00	0.45
R02	−0.61		1.00	1.00	1.00	1.00	1.00	1.00	1.00	1.00	1.00	1.00	0.97
R04	0.65	0.49		1.00	1.00	1.00	1.00	1.00	1.00	1.00	1.00	1.00	0.99
R08	−0.44	1.20	0.18		1.00	1.00	1.00	1.00	1.00	1.00	1.00	1.00	0.95
R09	−1.29	−0.26	−1.24	−0.43		1.00	1.00	0.99	0.67	0.97	1.00	1.00	0.86
R14	0.69	−0.51	−1.16	−0.48	−0.87		1.00	1.00	1.00	1.00	1.00	1.00	0.81
R18	0.17	0.22	0.65	0.10	0.10	0.02		1.00	1.00	1.00	0.97	0.99	0.45
R19	−1.32	−0.75	−1.34	−1.10	0.76	1.03	−0.67		0.73	1.00	1.00	1.00	0.72
JB02	2.45	−0.04	−0.89	−0.39	0.88	0.95	1.35	−0.70		−0.17	0.16	0.98	−0.04
JB03	0.67	−0.61	0.07	−0.32	−1.06	−0.02	0.19	−1.94	−1.40		0.63	1.00	0.64
JB04	−0.24	−0.13	−1.05	−0.33	−0.64	1.49	1.15	−1.99	−0.96	−0.34		0.82	−0.93
JB05	−0.60	−0.41	−1.03	−0.01	−0.85	−1.06	−0.60	−1.26	−0.75	−0.65	−0.07		0.46
R17	0.00	0.71	−1.58	−0.47	−1.18	0.22	1.37	−2.13	0.01	−1.40	−1.44	−1.30	

*Data at bottom left are βNTI values, while data at top right are RC_Bray_ values. Yellow, dispersal limitation; green, undominated process; purple, heterogeneous selection; blue, homogeneous selection*.

Secondly, the null-model-based taxonomic β-diversity metrics RC_Bray_ was used to further partition the pairwise comparisons governed by stochastic processes (|βNTI| <2) including dispersal, diversification and drift. According to Table 1, most RC_Bray_ values of the pairwise comparisons with |βNTI| <2 were >0.95 (α = 0.05 by a two-tailed test), indicating the given pairs shared significantly less similarity of community structure than the null expectation. Thus, dispersal limitation, which would lead to less similar communities, was quantified as the dominant fraction of the pairwise comparisons with |βNTI| <2 and RC_Bray_ >0.95.

At last, the pairwise comparisons with |βNTI| <2 and |RC_Bray_| <0.95 were inferentially controlled by “undominated” fraction, which was mostly consisted of diversification and/or drift and could not be further partitioned at this moment.

The assembly processes across all pairwise combinations were listed in Table 1. In inshore group, the community turnovers between pairwise sediments were all attributed to dispersal limitation. In offshore group, only 20% of the turnovers were explained by dispersal limitation, whereas 80% of the between-community compositional differences were “undominated.” Between the two groups, 77.5% of the turnovers were attributed to dispersal limitation, 17.5% to “undominated” fractions and 5% to deterministic selection. In all 13 Ross Sea sediments, 78.2% of the compositional turnovers were driven by dispersal limitation, 19.2% by “undominated” scenario, whereas deterministic selection only controlled 2.6% of the compositional turnovers.

### Influence of Environmental Variables

Spearman's rank correlation analysis was performed to assess the relationships between environmental variables and α-diversity indices (Table 2). Bacterial richness (Chao1 and ACE indices) and species diversity (Shannon and Simpson indices) were both positively correlated with temperature, chlorophyll and CDOM. While salinity, depth and pH didn't seem to be significantly correlated with alpha diversity.

**Table 2:** Correlation analysis between environmental variables and bacteria community.

**Factor**	**Spearman's correlation**				**Mantel test**	
	**Chao1**	**ACE**	**Shannon**	**Simpson**	**Jaccard**	**Bray-Curtis**
Salinity	0.48	0.53	0.50	0.45	0.50^**^	0.11
Temperature	0.79^**^	0.81^***^	0.77^**^	0.79^**^	0.74^***^	0.60^***^
Chlorophyll	0.75^**^	0.76^**^	0.71^**^	0.67*	0.58^**^	0.54^**^
CDOM	0.74^**^	0.76^**^	0.79^**^	0.76^**^	0.33^*^	0.23^*^
Depth	0.51	0.45	0.45	0.43	0.03	0.04
pH	0.08	0.09	0.41	0.37	0.07	−0.12
Geographic distance	–	–	–	–	0.46^**^	0.33^*^

* p < 0.05;

** p < 0.01;

*** *p < 0.001*.

According to Mantel test (Table 2), temperature, chlorophyll and CDOM were significantly correlated with bacterial community structure. Geographic distance between sampling stations also showed significant correlation with bacterial community structure, which evidenced the foregoing conclusion drawn from the null-model-based framework on the other side.

### Comparison of Culture-Dependent and -Independent Approaches

Conventional spread-plate method was used to isolate culturable bacteria. In total, 629 bacterial isolates were obtained from 13 sediment samples (Table S6). The taxonomic identification based on 16S rRNA genes assigned the 629 isolates to 4 phyla (*Proteobacteria* 73.61%, *Bacteroidetes* 17.81%, *Actinobacteria* 5.09% and *Firmicutes* 3.50%), 6 classes (*Gammaproteobacteria* 41.81%, *Alphaproteobacteria* 31.80%, *Flavobacteriia* 11.29%, *Cytophagales* 6.52%, *Actinobacteria* 5.09%, and *Bacilli* 3.50%), 14 orders, 26 families and 40 established genera. At the genus level, the abundant taxa included *Halomonas* (22.22%), *Marinobacter* (20.20%), *Pseudomonas* (7.58%), *Salegentibacter* (6.06%), *Salinibacterium* (5.30%), *Arenibacter* (4.55%), *Idiomarina* (4.55%), *Thalassospira* (4.29%), *Bacillus* (3.79%), *Gillisia* (3.03%), *Martelella* (2.02%), *Methylophaga* (1.52%), *Planomicrobium* (1.26%), *Pseudidiomarina* (1.26%), *Roseivirga* (1.26%), and *Loktanella* (1.01%).

**Figure 5:**
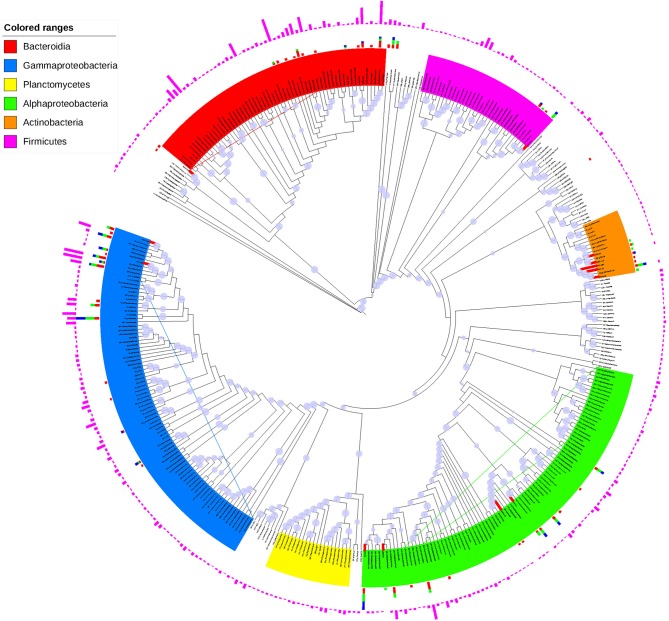
ML tree based on representative sequences of OTUs generated by CD-HIT-EST. The slate-blue pies on the clade lines represent bootstrap values (70%). The red and bold clades indicate OTUs only detected by culture-dependent method. The pink, red, green, and blue charts out of the OTU labels showed the square-rooted sequence numbers detected by high-throughput sequencing, MA, MC, and LC plates. The lowercase letters between OTU number and taxonomical name: c, class; o, order; f, family; g, genus.

With a sequence identity cutoff of 97%, the 629 isolates obtained from conventional spread-plate method were clustered into 60 OTUs, 47 of which were also discovered by culture-independent technique (Figure S6). Interestingly, there were 13 OTUs of isolated bacteria, including *Actinobacteria, Alphaproteobacteria, Cytophagales, Bacillus, Pseudomonas*, and *Marinobacter*, were not detected by culture-independent technique (Table S7). When shown in an ML tree (Figure 5), the bacteria isolates were mainly distributed in *Gammaproteobacteria, Alphaproteobacteria, Cytophaga*-*Flavobacteria*-*Bacteroides* group, *Firmicutes*, and *Actinobacteria*. In general, the high abundant OTUs of bacterial isolates also showed high abundance in OTU table generated by high-throughput sequencing, such as JB01-H26/OTU29 (*Halomonas*), R14-M19/OTU299 (*Marinobacter*), JB01-H16B/OTU24 (*Marinobacter*), JB01-H12/OTU45 (*Sphingomonadales*), R14-H23/OTU304 (*Pseudomonadaceae*), JB01-H9/OTU34 (*Rhodobacteraceae*), and JB01-H10/OTU69 (*Salegentibacter*) (Table 3 and Figure S7). However, there were also some OTUs of bacterial isolates showed significantly higher abundance than revealed by high-throughput sequencing, such as R18-M15/OTU121 (*Salinibacterium*), R14-M6/OTU84 (*Aurantimonadaceae*), R18H24/OTU104 (*Pseudomonas*), R04-H28/OTU86 (*Brevundimonas*), and OTU113 (*Zhouia*). Most of the culture-dependent OTUs could be isolated on MA plates, whereas 7 out of 60 OTUs could only be isolated by MC and/or LC plates.

**Table 3:** Top 10 OTUs of isolates with highest abundance and their taxonomy.

**OTU of isolates**	**Abundance (%)**	**Culture-independent OTU**	**Abundance (%)**	**Taxonomy**
JB01-H26	13.99	OTU29	1.16	p__Proteobacteria; c__Gammaproteobacteria; o__Oceanospirillales; f__Halomonadaceae; g__Halomonas
R14-L2	11.45	ND	0.00	p__Proteobacteria; c__Alphaproteobacteria; o__Rhodobacterales; f__Rhodobacteraceae; g__
R14-M19	6.84	OTU299	1.84	p__Proteobacteria; c__Gammaproteobacteria; o__Alteromonadales; f__Alteromonadaceae; g__Marinobacter
JB01-H16B	4.93	OTU24	5.85	p__Proteobacteria; c__Gammaproteobacteria; o__Alteromonadales; f__Alteromonadaceae; g__Marinobacter
JB01-H12	4.77	OTU45	3.53	p__Proteobacteria; c__Alphaproteobacteria; o__Sphingomonadales; f__; g__
R14-H23	4.61	OTU304	0.17	p__Proteobacteria; c__Gammaproteobacteria; o__Pseudomonadales; f__Pseudomonadaceae; g__
JB01-H9	3.97	OTU34	1.21	p__Proteobacteria; c__Alphaproteobacteria; o__Rhodobacterales; f__Rhodobacteraceae; g__
JB01-H10	3.82	OTU69	8.60	p__Bacteroidetes; c__Flavobacteriia; o__Flavobacteriales; f__Flavobacteriaceae; g__Salegentibacter
R18-M15	3.34	OTU121	0.03	p__Actinobacteria; c__Actinobacteria; o__Actinomycetales; f__Microbacteriaceae; g__Salinibacterium
JB03-H13	3.18	OTU6	0.31	p__Bacteroidetes; c__Cytophagia; o__Cytophagales; f__Cyclobacteriaceae; g__

*ND, not detected by culture-independent technique*.

## Discussion

The bacterial community in Antarctic marine benthic ecosystems were barely studied, compared with in terrestrial habitats, sea ice and seawater of Antarctica. Especially in higher latitude Antarctic continental shelf, only few sediments from coastal shallows and individual stations were studied for bacterial diversity and community composition (Carr et al., 2013; Learman et al., 2016). The present study featured a biological hotspot area in high-latitude Antarctic continental shelf, extending from the northern border to the southern border and from coast to the Central Trough of the Ross Sea, in order to provide comprehensive insights into the community structure, metabolic and ecological mechanisms, assembly processes of the bacterial community and the influence of environmental variables.

The bacterial richness and diversity in 13 sediments of the Ross Sea were lower than in the sediments beneath the Ross Ice Shelf (Carr et al., 2013), along the Antarctic Polar Front (Ruff et al., 2014) and the western coast of the Ross Sea (Learman et al., 2016), and across the western Arctic Ocean (Dong et al., 2017). This discrepancy was possibly attributable to the different OTU-generating algorithms. QIIME or Mothur used in the mentioned references above were reported to generate more OTUs than UPARSE used in this study (Callahan et al., 2016).

The most abundant and diverse taxa in sediments of the Ross Sea were *Bacteroidetes, Gamma* and *Alphaproteobacteria*, which also dominated other various marine and terrestrial habitats in polar regions (Carr et al., 2013, 2015; Dong et al., 2017; Franco et al., 2017; Wang et al., 2017), as well as temperate and tropical marine environments (Kumbhare et al., 2015; Xie et al., 2017). Whereas, terrestrial habits in temperate and tropical areas were generally dominated by *Actinobacteria, Alpha-, Beta-* and *Gammaproteobacteria, Acidobacteria*, and *Planctomycetes* (Malard and Pearce, 2018). Thus, we conclude that the divergence of bacterial community composition between marine and terrestrial ecosystems in polar regions was not as great as in temperate and tropical zones, possibly because of that the harsh conditions in polar terrestrial environments, such as low temperature, ice-covering, low nutrient and without vegetation cover, shaped similar bacterial communities with in marine environments (Boetius et al., 2015). *Bacteroidetes, Gamma-* and *Alphaproteobacteria* were reported to have adaptive mechanisms in such harsh environments (Hamdan et al., 2013; Williams and Cavicchioli, 2014).

The bacterial community in the Ross Sea sediments has developed adaptive mechanisms to environmental stresses including oligotrophy, anoxia, and low temperature. The aerobic chemoheterotrophs displayed competitive relationships in network analysis (Figure 4), emphasizing the scarcity of nutrients and oxygen in the Ross Sea sediments. Hence some benthic bacteria had ability of utilizing refractory carbon, such as xylan, cellulose, chitin, hydrocarbon, aromatic compounds, plastics, and even methyl compounds (Table S5). Members of *Gammaproteobacteria* and *Cytophaga-Flavobacteria-Bacteroides* group were reported to be associated with algal blooms and capable of degrading complex polysaccharides (Bowman et al., 2003; Wietz et al., 2015; Franco et al., 2017). Indigenous hydrocarbon-degrading bacteria, such as *Alcanivorax, Marinobacter, Sphingomonas*, and *Rhodococcus*, were able to utilize alkanes and/or polycyclic aromatic hydrocarbons produced by marine algae, natural hydrocarbon seeps and anthropogenic activities in marine ecosystems (Wang and Shao, 2012; Wang et al., 2014). Recently, artificial oil spills and microplastic pollutions were detected in Antarctic continental shelves, and their impacts on ecosystems are fascinating (Brown et al., 2016; Reed et al., 2018). *Methylococcales* was a key component of consuming methane and other C_1_ compounds to produce organics and energy (Ruff et al., 2019). Because of anoxia, some bacteria in sediments of the Ross Sea performed dark sulfur oxidation and manganese oxidation to derive energy, and used nitrate, nitrite, sulfate, thiosulfate, fumarate, and arsenate as electron acceptors (Ulrich and Kretzschmar, 2007; Fike et al., 2016). Sulfur oxidation bacteria were likely to play an important role in carbon sequestration, since half of the total dark carbon fixation in the oceans was sulfur-dependent, occurred in marine sediments, and mostly driven by uncultured *Gammaproteobacteria* (Dyksma et al., 2016). Anaerobic bacteria formed a stable, compact and conserved module (Figure 4). The positive correlations to each other within this module were possibly contributed to their common requirement of anaerobic niches. Besides obligate psychrophiles, such as *Psychroserpens, Psychroflexus, Psychrobacter*, some members of *Marinobacter, Halomonas, Gillisia, Pseudomonas*, and *Flavobacteriaceae* were also reported to show adaptive characteristics to low temperature, such as production of exopolysaccharides, cold active enzymes and pigments, and changes in membrane lipid composition and fluidity (Fan et al., 2013; Kumbhare et al., 2015).

How did this characteristic bacterial community form? Although the debate between niche and neutral theory is one of the central issues of community ecology in last a couple of decades, recent conclusions deemed that both deterministic and stochastic processes jointly drive the assembly of communities (Cira et al., 2018). Whereas, a further debate is on the relative importance of deterministic and stochastic processes on shaping community structure, succession and biogeography (Zhou and Ning, 2017). Recently, a well-accepted quantitative framework has been developed to disentangle and quantify the influences of various ecological processes (Stegen et al., 2013). Results of this null-model-based framework suggested that the most important ecological forces driving community assembly in sediments of the Ross Sea were dispersal limitation, in consist with the conclusion revealed by Mantel test that geographic distance was correlated with bacterial community structure. Dispersal limitation and undominated fractions were inferred to be predominantly stochastic (Vellend, 2010; Stegen et al., 2013; Zhou and Ning, 2017). Therefore, our results suggested that stochastic processes appear more important than deterministic ones for community assembly in sediments of the Ross Sea. To our best knowledge, no studies have been performed as yet to investigate the community assembly mechanism in polar marine ecosystems. More interestingly, many literatures proposed that deterministic and stochastic processes gained dominated importance under stressful and probiotic environments, respectively (Lepori and Malmqvist, 2009; Chase, 2010; Kim, 2019). Nevertheless, our results suggested that stochastic processes would also be dominated in some stressful environments, such as Antarctic continental shelves.

On the other hand, the deterministic process also played non-negligible roles in shaping community structure. Correlation analysis and Mantel test suggested that bacterial richness, diversity and structure were significantly influenced by CDOM, temperature and chlorophyll. Since majority of the bacteria detected in this study were chemoheterotrophs, it was logical that CDOM positively correlated with bacterial richness and diversity. Temperature, organic matter and chlorophyll were frequently reported to account for variability of bacterial communities in polar marine sediments and other deep sea and benthic environments (Learman et al., 2016; Franco et al., 2017; Wang et al., 2017; Medina-Silva et al., 2018).

In consistent with some other researches, this study found that bacterial communities in inshore sediments showed higher species richness and α-diversity than in offshore sediments (Semprucci et al., 2010; Liu et al., 2018). One explanation is that inshore sediments have higher concentration of organic matter, which was suggested as immediate factor determining the richness and diversity of bacteria communities in Antarctic marine sediments (Carr et al., 2013; Franco et al., 2017). It was reported that coastal areas of maritime Antarctica had higher nutrient concentration because of nutrient input from terrestrial runoffs (Izaguirre et al., 2003). High nutrients in coastal areas supported the blooms of phytoplankton, bacterioplankton and microphytobenthos, and induced higher primary production and more organic matter both in upper water column and in sediments (Ardyna et al., 2017; Hill-Spanik et al., 2019).

The present study found that temperature was positively correlated with chlorophyll and CDOM, and chlorophyll was positively correlated with CDOM. In the climate warming scenario, temperature elevation significantly increased the release of land-derived materials into the aquatic environments, the uptake of inorganic nutrients, the chlorophyll concentration, the primary production, and subsequently the concentration of organic matter in marine sediments (Cormier et al., 2016; Fountain et al., 2016; Sultana et al., 2016; Ardyna et al., 2017; Tew et al., 2017; Spackeen et al., 2019). Polar regions are warming much faster than temperate and tropical areas because of reduction of snow cover, lack of vegetation layer, sea ice loss, and variation of atmospheric and ocean circulation (Fountain et al., 2016; D'Angelo et al., 2018). Therefore, further evaluating the impact of global warming on microflorae in polar environments is especially important.

Isolating and characterizing microorganisms could provide insights into their phylogenetic systematics, metabolic reactions and physiological properties, and help to understand the formation, persistence, adaptation mechanisms and ecological functions of microbial community. Moreover, Antarctic continental shelf is a fertile ground for bioprospecting, attracting increasing attention and research efforts recently (Van Trappen et al., 2002; Lewin et al., 2013; Vester et al., 2015). The exploitation of Antarctic microorganisms and gene pool would offer functional products, such as cold-active enzymes, anti-freeze proteins, pigments, antibiotics, dietary supplements (for instance polyunsaturated fatty acids) and bioremediation technologies (Van Trappen et al., 2002; Bowman et al., 2003; Kim et al., 2010; De Pascale et al., 2012; Giudice and Fani, 2015). In the present study, spread-plate method was used as parallel complement of culture-independent approach and yielded 629 isolates, which would be helpful to perform more metabolic, physiological and ecological tests in future. Our previous studies have phenotypically, phylogenetically and chemotaxonomically characterized five novel type strains out of the 629 isolates (Li et al., 2017a,b, 2018a,b,c).

Compared with culture-dependent techniques which had limitations because of the selectivity of isolation media and culture conditions, sequencing-based molecular techniques were considered to provide better resolution of microbial community composition, however, it still showed biases. Although the Good's coverage values and rarefaction curves indicated that overwhelming majority of bacteria were revealed from the sediments, there were still 13 OTUs only detected by culture-dependent technique (Figure S6, Table S7). Most of the OTUs preferring culture-dependent technique (Tables S7, S8) belonged to *Actinobacteria, Alphaproteobacteria*, and *Bacillales*, which were reported to be underestimated during high-throughput sequencing (Guo and Zhang, 2013; Velasquez-Mejia et al., 2018). On the other hand, although *Pseudomonas* and *Marinobacter* were abundant taxa as revealed by both techniques, *Pseudomonas* sp. JB01-H21 and *Marinobacter* sp. R17-H24 were not detected by high-throughput sequencing based on 16S rRNA gene amplicons. Due to the huge number, small size and widely distribution of microbes in environment, it has been proved that the conventional sampling process and high-throughput sequencing of 16S rRNA gene amplicons would dramatically underestimated the bacterial diversity, especially for the rare community members (Zhou et al., 2013; Meyer et al., 2018). Therefore, combining culture-dependent and independent approaches would be a better option to research the microbial community (Aislabie et al., 2006; Fan et al., 2013).

In summary, the present study provided detailed insights into the diversity, structure and assembly mechanisms of the benthic bacterial community in the Ross Sea, using both culture-dependent and -independent approaches. The bacterial community was dominated by *Bacteroidetes, Gamma-* and *Alphaproteobacteria*, which are abundant and widely distributed in worldwide seawaters and marine sediments. Previous studies focused on the effect of environmental factors on bacterial community of maritime Antarctica (Carr et al., 2013; Jamieson et al., 2013; Learman et al., 2016). However, although environmental factors, mainly organic matter, chlorophyll and temperature, were found to be significantly correlated with the community richness, diversity and structure, our results proved that stochastic processes were the main forces driving community assembly. Therefore, it is interesting to further study the trade-off between stochastic and deterministic processes in shaping the microbial community on Antarctic continental shelves. Our study was helpful to understand the structural and functional aspects, as well as the ecological role and biogeochemical processes of the benthic community in the Ross Sea, an important biological hotspot in high-latitude Antarctic areas which is undergoing a rapid climate change and possible community shifts.

## Data Availability Statement

The raw data obtained by high-throughput sequencing of 16S rRNA gene amplicons in this study were deposited in NCBI Sequence Read Archive database under the accession numbers of SRR8505880 to SRR8505892. The 16S rRNA gene sequences of 629 isolates were submitted to NCBI GenBank database under the accession numbers of MK476530 to MK477158.

## Author Contributions

H-HZ and QY designed the study and reviewed the manuscript prior to submission. X-BH collected the sediment samples and geochemical data. M-XZ and MC processed the samples for sequencing. A-ZL performed the metagenomics analysis, generated all tables and figures, and wrote the draft manuscript. A-ZL and YZ performed the statistical analysis.

### Conflict of Interest

The authors declare that the research was conducted in the absence of any commercial or financial relationships that could be construed as a potential conflict of interest.
