# Supplementary material for: Culture-Dependent and -Independent Analyses Reveal the Diversity, Structure, and Assembly Mechanism of Benthic Bacterial Community in the Ross Sea, Antarctica
Source: Front Microbiol. 2019 Nov 8;10:2523. doi: 10.3389/fmicb.2019.02523 (PMC6856632; doi:10.3389/fmicb.2019.02523)
Supplement: Supplementary file 1 [file Data_Sheet_1.pdf]

Table S1 Geographical and chemical characteristics of sediment samples collected from the Ross Sea.

Group	Sample	Longitude	Latitude	Salinity (PSU)	Temperature (ºC)	Chlorophyll (μg/L)	CDOM (ppb)	Depth (m)	pH
Inshore	JB01	165.5694	-77.5883	33.84	3.15	5.81	2.01	774	7.96
	R02	165.133	-74.785	33.93	3.36	5.51	2.39	719	8.29
	R04	166.7767	-74.7667	34.05	0.35	1.18	1.85	901	7.95
	R08	165.012	-75.003	34.12	3.08	7.15	2.01	892	8.77
	R09	165.998	-75.0033	33.53	2.87	6.01	2.01	1032	8.04
	R14	164.8047	-74.935	34.13	3.58	7.26	1.93	901	8.28
	R18	163.765	-74.913	34.23	3.48	6.39	2.16	46	8.49
	R19	170.3944	-72.2544	33.83	3.34	5.14	2.08	516	9.05
	(mean)	-	-	33.96	2.90	5.56	2.06	723	8.35
Offshore	JB02	169.9867	-76.8442	34.13	1.05	2.18	1.77	760	8.61
	JB03	170.6983	-75.8036	33.33	0.53	1.72	1.77	615	7.65
	JB04	172.3725	-75.3011	33.71	0.88	2.09	1.77	511	7.99
	JB05	173.1881	-74.7553	33.46	0.67	1.8	1.77	497	7.58
	R17	167.9092	-75.2303	32.14	-0.33	1.71	1.85	374	8.65
	(mean)	-	-	33.35	0.56	1.90	1.79	551	8.10

Table S2 Spearman's rank correlation analysis between environmental variables of the sediment samples collected from the Ross Sea. Data at bottom left are *r* values; data at top right are *p* values.

Variable	Salinity	Temperature	Chlorophyll	CDOM	Depth	pH
Salinity		0.028	0.099	0.287	0.404	0.765
Temperature	0.61		5.08E-07	0.002	0.635	0.218
Chlorophyll	0.48	0.95		0.009	0.527	0.184
CDOM	0.32	0.77	0.69		0.860	0.204
Depth	0.25	0.15	0.19	-0.05		0.617
pH	0.09	0.37	0.39	0.38	-0.15	

Table S3 Sequencing processing and estimates of alpha diversity of the sediment samples collected from the Ross Sea. Welch's t-tests of Chao1, ACE, Shannon and Simpson indices between the two groups was performed by R.

Group	Sample	Valid reads	Qualified sequences	Coverage (%)	OTUs	Chao1	ACE	Shannon	Simpson
Inshore	JB01	32857	19222	99.82	228	232.84	233.74	5.06	0.94
	R02	29167	17790	99.81	199	292.00	283.80	5.75	0.96
	R04	36375	22164	99.86	187	183.23	179.27	3.40	0.79
	R08	36357	21976	99.83	265	219.53	221.55	5.25	0.93
	R09	35336	23034	99.82	152	203.07	193.08	4.52	0.91
	R14	36678	22599	99.81	220	253.00	241.00	4.93	0.93
	R18	37262	23449	99.89	186	191.47	193.67	4.42	0.87
	R19	35476	22889	99.86	188	190.06	189.46	5.17	0.95
	(mean)	34939	21640	99.84	203	220.65	216.95	4.82	0.91
Offshore	JB02	20998	13710	99.84	94	109.40	114.63	3.57	0.87
	JB03	37016	23533	99.89	122	127.00	126.99	3.01	0.79
	JB04	37159	24088	99.9	91	94.09	98.73	2.76	0.77
	R17	36449	24266	99.93	43	42.33	42.42	1.91	0.53
	JB05	38899	26243	99.89	136	136.91	142.64	2.84	0.77
	(mean)	34104	22368	99.89	97	101.95	105.08	2.82	0.74
p-value	-	-	-	-	-	<2e-6	<7e-7	<5e-8	<8e-12

Table S4 ANCOM test results for differential abundance of bacterial taxa at the family and genus level. High W values indicate significant differences in abundance levels between inshore and offshore group. Higher W value indicates more significant difference in abundance level. The ANCOM analysis was achieved by QIIME2.

Percentile	0	25	50	75	100	0	25	50	75	100	Reject null hypothesis	W
Group	Inshore					Offshore						
f_Alcaligenaceae	1.07	1.18	1.33	1.37	1.42	1.00	1.00	1.02	1.02	1.06	FALSE	2
f_Alcanivoracaceae	1.32	4.98	7.59	10.51	15.89	1.00	1.00	1.00	1.02	1.04	FALSE	8
f_Alteromonadaceae	1.42	15.11	35.06	41.89	54.86	1.50	14.60	51.70	69.75	73.89	FALSE	0
f_Aurantimonadaceae	1.01	1.05	1.13	1.15	1.54	1.00	1.00	1.00	1.07	1.15	FALSE	1
f_Bacillaceae	1.00	1.01	1.01	1.03	1.16	1.01	1.01	1.04	1.16	1.22	FALSE	2
f_Bacteroidaceae	4.58	9.97	18.77	35.01	118.35	2.14	4.07	14.01	22.69	23.21	FALSE	0
f_Balneolaceae	1.00	1.09	1.26	3.77	11.64	1.00	1.00	1.00	1.01	1.02	FALSE	0
f_Barnesiellaceae	1.00	1.01	1.04	1.10	1.26	1.00	1.00	1.01	1.02	1.04	FALSE	2
f_Bdellovibrionaceae	1.00	1.00	1.00	1.07	1.15	1.00	1.00	1.00	1.00	1.00	FALSE	1
f_Brucellaceae	1.00	1.00	1.00	1.00	1.03	1.00	1.00	1.00	1.00	1.00	FALSE	0
f_Burkholderiaceae	1.04	1.05	1.14	1.23	2.55	1.03	1.09	1.21	1.56	1.78	FALSE	1
f_Caulobacteraceae	1.01	1.03	1.18	1.38	3.07	1.00	1.00	1.04	1.21	3.89	FALSE	0
f_Chitinophagaceae	1.00	1.00	1.01	1.98	5.02	1.00	1.00	1.00	1.00	1.01	FALSE	0
f_Chromatiaceae	1.00	1.00	1.00	1.04	2.72	1.00	1.00	1.00	1.00	1.00	FALSE	0
f_Clostridiaceae	1.00	1.02	1.09	1.32	2.99	1.00	1.00	1.00	1.00	1.00	FALSE	1
f_Comamonadaceae	1.01	1.03	1.06	1.09	1.56	1.00	1.03	1.04	1.09	1.48	FALSE	0
f_Cryomorphaceae	1.00	1.00	1.00	1.21	2.57	1.00	1.00	1.00	1.00	1.00	FALSE	0

f_Cyclobacteriaceae	1.01	1.15	2.33	3.96	6.42	1.00	1.00	1.00	1.00	1.00	FALSE	1
f_Cytophagaceae	1.00	1.00	1.00	1.08	3.25	1.00	1.00	1.00	1.00	1.00	FALSE	0
f_Desulfobacteraceae	1.00	1.01	1.07	1.19	1.92	1.00	1.00	1.02	1.04	1.04	FALSE	0
f_Desulfobulbaceae	1.00	1.00	1.00	1.11	1.22	1.00	1.00	1.00	1.01	1.02	FALSE	0
f_Desulfuromonadaceae	1.00	1.00	1.03	1.13	1.71	1.00	1.00	1.04	1.04	1.05	FALSE	0
f_Dietziaceae	1.00	1.00	1.03	1.10	1.27	1.00	1.00	1.00	1.00	1.01	FALSE	1
f_Enterobacteriaceae	1.32	1.48	2.21	3.13	17.04	1.15	1.40	2.54	4.13	8.21	FALSE	0
f_Erysipelotrichaceae	1.01	1.04	1.10	1.18	1.59	1.00	1.00	1.05	1.06	1.10	FALSE	2
f_Erythrobacteraceae	1.05	1.68	2.45	7.43	17.50	1.00	1.00	1.00	1.88	1.88	FALSE	0
f_Flammeovirgaceae	1.11	2.04	3.99	5.84	7.21	1.00	1.00	1.00	1.00	1.02	FALSE	2
f_Flavobacteriaceae	2.34	15.09	40.41	83.44	92.46	59.77	102.94	121.65	128.26	169.51	FALSE	4
f_Fusobacteriaceae	1.77	2.21	4.56	6.75	25.04	1.14	1.70	3.99	5.10	5.60	FALSE	0
f_Halomonadaceae	1.12	1.33	3.04	5.56	7.25	4.25	4.36	5.92	9.82	95.10	FALSE	0
f_Hyphomicrobiaceae	1.07	1.23	1.90	3.01	6.52	1.01	1.06	1.22	1.36	1.91	FALSE	0
f_Hyphomonadaceae	1.01	1.30	3.51	4.70	6.75	1.00	1.00	1.00	1.00	1.02	FALSE	2
f_Idiomarinaceae	1.03	2.19	5.30	8.69	33.96	1.00	1.02	1.07	1.79	21.41	FALSE	0
f_Ignavibacteriaceae	1.00	1.00	1.02	1.06	1.19	1.00	1.00	1.00	1.01	1.03	FALSE	1
f_Kiloniellaceae	1.02	1.11	1.20	1.42	8.35	1.00	1.00	1.00	1.00	1.01	FALSE	0
f_Kordiimonadaceae	1.00	1.02	1.05	1.20	1.97	1.00	1.00	1.00	1.00	1.00	FALSE	0
f_Lachnospiraceae	1.65	2.14	4.07	5.72	17.93	1.12	1.35	3.17	4.55	4.85	FALSE	1
f_Marinicellaceae	1.00	1.02	1.11	1.63	2.45	1.00	1.02	1.07	1.12	1.27	FALSE	0
f_Marinilabiaceae	1.00	1.00	1.00	1.00	2.85	1.00	1.00	1.00	1.00	1.00	FALSE	0
f_Methylobacteriaceae	1.00	1.00	1.01	1.03	1.07	1.00	1.00	1.01	1.02	1.02	FALSE	1
f_Microbacteriaceae	1.00	1.01	1.02	1.06	1.15	1.00	1.00	1.00	1.00	1.52	FALSE	0
f_Moraxellaceae	1.01	1.09	1.23	1.42	1.68	2.99	4.43	8.28	15.23	34.05	TRUE	496
f_Neisseriaceae	1.00	1.00	1.02	1.03	1.10	1.00	1.00	1.00	1.01	1.01	FALSE	1

f_Nitrosomonadaceae	1.00	1.01	1.05	1.21	1.45	1.00	1.02	1.03	1.05	1.23	FALSE	0
f_Nocardioidaceae	1.00	1.00	1.00	1.00	1.01	1.00	1.00	1.00	1.00	1.71	FALSE	0
f_Oceanospirillaceae	1.00	1.00	1.00	1.00	1.00	1.00	1.00	1.00	1.00	1.17	FALSE	0
f_Opitutaceae	1.02	1.08	1.15	1.34	1.52	1.00	1.00	1.00	1.00	1.00	FALSE	2
f_Oxalobacteraceae	1.07	1.14	1.26	1.38	4.26	1.03	1.11	1.51	1.86	2.56	FALSE	0
f_Paraprevotellaceae	1.02	1.09	1.13	1.22	1.69	1.00	1.02	1.08	1.12	1.14	FALSE	2
f_Phycisphaeraceae	1.03	1.17	1.53	2.37	7.70	1.00	1.00	1.00	1.00	1.00	FALSE	0
f_Phyllobacteriaceae	1.03	1.65	1.97	3.11	3.70	1.00	1.00	1.00	1.00	1.01	FALSE	3
f_Pirellulaceae	1.02	1.06	1.23	1.73	3.30	1.00	1.00	1.00	1.01	1.02	FALSE	0
f_Piscirickettsiaceae	1.39	2.42	2.82	5.01	16.51	1.06	1.27	1.29	1.31	1.33	FALSE	1
f_Planctomycetaceae	1.00	1.15	1.36	1.68	2.33	1.00	1.00	1.00	1.00	1.01	FALSE	2
f_Planococcaceae	1.00	1.00	1.02	1.11	2.03	1.00	1.00	1.03	1.10	1.18	FALSE	0
f_Porphyromonadaceae	1.03	1.05	1.08	1.15	1.60	1.00	1.03	1.06	1.16	1.17	FALSE	2
f_Pseudoalteromonadaceae	1.00	1.01	1.02	1.03	1.21	1.01	1.04	1.04	3.07	4.21	FALSE	0
f_Pseudomonadaceae	1.08	1.17	2.56	3.53	6.65	1.80	11.08	17.45	19.45	27.52	FALSE	5
f_Rhodobacteraceae	1.48	3.75	4.46	8.67	12.62	1.01	1.91	2.73	7.35	9.03	FALSE	1
f_Rhodospirillaceae	1.29	1.62	1.85	2.96	11.57	1.00	1.00	1.00	1.00	1.02	FALSE	0
f_Rikenellaceae	1.00	1.02	1.06	1.17	1.51	1.01	1.01	1.01	1.02	1.11	FALSE	2
f_Ruminococcaceae	1.02	1.11	1.20	1.31	2.51	1.01	1.04	1.12	1.29	1.36	FALSE	2
f_Saprospiraceae	1.00	1.00	1.00	1.06	1.53	1.00	1.00	1.00	1.00	1.00	FALSE	0
f_Shewanellaceae	1.00	1.00	1.00	1.04	1.89	1.00	1.00	1.00	1.00	1.00	FALSE	0
f_Simkaniaceae	1.00	1.00	1.00	1.00	1.26	1.00	1.00	1.00	1.00	1.00	FALSE	0
f_Sinobacteraceae	1.00	1.01	1.05	1.09	1.28	1.00	1.00	1.00	1.00	1.01	FALSE	1
f_Sphingobacteriaceae	1.00	1.00	1.03	1.11	1.14	1.00	1.00	1.00	1.00	1.00	FALSE	1
f_Sphingomonadaceae	1.01	1.09	1.37	1.97	2.77	1.00	1.00	1.05	1.07	1.19	FALSE	1
f_Staphylococcaceae	1.00	1.01	1.02	1.03	1.08	1.00	1.00	1.01	1.01	1.02	FALSE	1

f_Streptococcaceae	1.00	1.00	1.01	1.02	1.07	1.00	1.00	1.00	1.00	1.04	FALSE	0
f_Syntrophobacteraceae	1.00	1.00	1.00	1.00	1.50	1.00	1.00	1.00	1.00	1.00	FALSE	0
f_Thermaceae	1.01	1.02	1.03	1.05	1.32	1.00	1.01	1.03	1.07	1.21	FALSE	0
f_Thermodesulfovibrionaceae	1.00	1.00	1.02	1.07	1.34	1.00	1.01	1.04	1.09	1.11	FALSE	0
f_Thiotrichaceae	1.00	1.00	1.01	1.97	53.58	1.00	1.00	1.00	1.00	1.01	FALSE	0
f_Veillonellaceae	1.08	1.14	1.29	1.52	3.60	1.00	1.05	1.28	1.38	1.39	FALSE	1
f_Xanthomonadaceae	1.00	1.00	1.00	1.02	1.27	1.00	1.03	1.04	1.04	1.11	FALSE	0
g_Acinetobacter	1.00	1.00	1.00	1.20	1.56	1.00	1.00	1.14	1.30	1.32	FALSE	2
g_Aequorivita	1.00	1.00	1.00	1.00	3.24	1.00	1.00	1.00	1.00	1.00	FALSE	0
g_Aeromicrobium	1.00	1.00	1.00	1.00	1.00	1.00	1.00	1.00	1.00	1.86	FALSE	0
g_Alcanivorax	1.24	2.55	3.33	4.15	5.47	1.00	1.00	1.00	1.00	1.00	FALSE	4
g_Algoriphagus	1.00	1.00	1.00	1.00	2.25	1.00	1.00	1.00	1.00	1.00	FALSE	0
g_Alishewanella	1.00	1.00	1.00	1.00	2.16	1.00	1.00	1.00	1.00	1.00	FALSE	0
g_Arenibacter	1.00	2.59	3.61	4.77	14.36	1.00	1.00	1.00	1.00	1.00	FALSE	8
g_Bacteroides	2.92	4.86	8.84	15.67	56.99	1.61	2.34	8.40	10.09	11.11	FALSE	0
g_Balneola	1.00	1.00	1.00	1.47	3.90	1.00	1.00	1.00	1.00	1.00	FALSE	0
g_Bdellovibrio	1.00	1.00	1.00	1.00	1.12	1.00	1.00	1.00	1.00	1.00	FALSE	0
g_Bizionia	1.00	1.00	1.00	1.00	1.00	1.00	1.49	1.59	39.95	81.93	FALSE	0
g_Burkholderia	1.00	1.00	1.00	1.13	2.26	1.00	1.00	1.00	1.00	1.32	FALSE	1
g_Caulobacter	1.00	1.00	1.00	1.00	1.21	1.00	1.00	1.00	1.00	1.00	FALSE	0
g_Clostridiisalibacter	1.00	1.00	1.00	1.03	1.22	1.00	1.00	1.00	1.00	1.00	FALSE	1
g_Cobetia	1.00	1.00	1.00	1.00	1.00	1.00	1.00	1.00	1.90	17.33	FALSE	0
g_Congregibacter	1.00	1.00	1.00	1.00	1.11	1.00	1.00	1.00	1.00	1.00	FALSE	0
g_Constrictibacter	1.00	1.00	1.00	1.06	3.02	1.00	1.00	1.00	1.00	1.00	FALSE	0
g_Desulfococcus	1.00	1.00	1.00	1.19	1.33	1.00	1.00	1.00	1.00	1.00	FALSE	1
g_Desulfotignum	1.00	1.00	1.00	1.00	1.65	1.00	1.00	1.00	1.00	1.00	FALSE	0

g_Devosia	1.00	1.00	1.00	1.03	1.58	1.00	1.00	1.00	1.00	1.00	FALSE	0
g_Dietzia	1.00	1.00	1.00	1.06	1.29	1.00	1.00	1.00	1.00	1.00	FALSE	1
g_Erythrobacter	1.00	1.00	1.00	1.28	2.19	1.00	1.00	1.00	1.62	1.63	FALSE	2
g_Escherichia	1.27	1.36	1.89	2.43	12.70	1.13	1.27	2.12	2.51	6.17	FALSE	0
g_Flavobacterium	1.00	1.00	1.00	1.00	1.50	1.00	1.00	1.00	1.00	1.00	FALSE	0
g_Fulvivirga	1.00	1.00	1.00	1.03	2.40	1.00	1.00	1.00	1.00	1.00	FALSE	0
g_Fusobacterium	1.00	1.71	3.21	4.45	12.87	1.00	1.50	2.72	3.37	4.07	FALSE	0
g_Gelidibacter	1.00	1.00	1.00	1.07	2.21	1.00	1.00	1.00	1.00	1.00	FALSE	1
g_Gillisia	1.00	1.00	1.54	7.14	15.17	1.00	1.00	2.07	8.97	18.37	FALSE	0
g_Glaciecola	1.00	1.00	1.00	1.03	1.18	1.00	1.00	1.00	1.00	1.00	FALSE	0
g_Gramella	1.00	1.00	1.00	1.34	2.07	1.00	1.00	1.00	1.00	1.00	FALSE	0
g_Halomonas	1.00	1.00	1.40	2.92	3.31	1.00	2.21	3.10	3.43	3.73	FALSE	0
g_Hyphomonas	1.00	1.00	1.14	1.20	1.51	1.00	1.00	1.00	1.00	1.00	FALSE	2
g_Idiomarina	1.00	1.00	1.00	2.25	2.98	1.00	1.00	1.00	1.55	5.11	FALSE	0
g_Inquilinus	1.00	1.00	1.00	1.00	1.22	1.00	1.00	1.00	1.00	1.00	FALSE	0
g_Lewinella	1.00	1.00	1.00	1.00	1.29	1.00	1.00	1.00	1.00	1.00	FALSE	0
g_Loktanella	1.00	1.00	1.16	1.55	3.62	1.00	1.21	1.37	1.48	3.92	FALSE	0
g_Lutimonas	1.00	1.00	1.00	1.00	1.63	1.00	1.00	1.00	1.00	1.00	FALSE	1
g_Maribacter	1.00	1.00	1.00	1.58	4.04	1.00	1.00	1.15	2.14	48.78	FALSE	0
g_Maricaulis	1.00	1.00	1.80	2.80	3.41	1.00	1.00	1.00	1.00	1.00	FALSE	1
g_Marinibacillus	1.00	1.00	1.00	1.00	1.11	1.00	1.00	1.00	1.00	1.00	FALSE	0
g_Marinobacter	1.00	5.82	11.09	15.10	18.83	1.47	6.02	19.13	24.69	38.10	FALSE	0
g_Mesonia	1.00	1.00	1.00	1.07	2.30	1.00	1.00	1.00	1.00	1.00	FALSE	0
g_Methylophaga	1.00	1.00	1.31	1.81	4.02	1.00	1.00	1.00	1.22	1.23	FALSE	0
g_Methylotenera	1.00	1.00	1.00	1.00	1.34	1.00	1.00	1.00	1.00	1.00	FALSE	0
g_Muricauda	1.00	1.00	1.10	1.90	3.94	1.00	1.00	1.00	1.00	1.00	FALSE	0

g_Mycoplana	1.00	1.00	1.00	1.00	1.00	1.00	1.00	1.00	1.00	2.02	FALSE	0
g_Nisaea	1.00	1.00	1.00	1.03	1.40	1.00	1.00	1.00	1.00	1.00	FALSE	2
g_Nitratireductor	1.00	1.00	1.00	1.00	1.42	1.00	1.00	1.00	1.00	1.00	FALSE	1
g_Nitriliruptor	1.00	1.00	1.00	1.00	1.10	1.00	1.00	1.00	1.00	1.00	FALSE	0
g_Nocardia	1.00	1.00	1.00	1.00	1.18	1.00	1.00	1.00	1.00	1.00	FALSE	0
g_Novosphingobium	1.00	1.00	1.00	1.19	2.34	1.00	1.00	1.00	1.00	1.00	FALSE	0
g_Olleya	1.00	1.00	1.00	1.12	1.48	1.00	1.00	1.00	1.00	1.00	FALSE	2
g_Opitutus	1.00	1.00	1.07	1.30	1.49	1.00	1.00	1.00	1.00	1.00	FALSE	1
g_Oscillospira	1.00	1.00	1.00	1.00	1.35	1.00	1.00	1.00	1.00	1.00	FALSE	0
g_Owenweeksia	1.00	1.00	1.00	1.14	2.25	1.00	1.00	1.00	1.00	1.00	FALSE	0
g_Paenisporosarcina	1.00	1.00	1.00	1.00	2.16	1.00	1.00	1.00	1.00	1.00	FALSE	0
g_Parabacteroides	1.00	1.00	1.00	1.00	1.43	1.00	1.00	1.00	1.00	1.00	FALSE	0
g_Paraprevotella	1.00	1.00	1.04	1.18	1.52	1.00	1.00	1.00	1.11	1.12	FALSE	2
g_Parvibaculum	1.00	1.00	1.00	1.38	2.05	1.00	1.00	1.00	1.00	1.00	FALSE	0
g_Phaeobacter	1.00	1.00	1.00	1.00	1.65	1.00	1.00	1.00	1.00	1.00	FALSE	0
g_Phascolarctobacterium	1.00	1.12	1.23	1.39	2.04	1.00	1.00	1.23	1.38	1.39	FALSE	2
g_Planctomyces	1.00	1.18	1.30	1.48	2.26	1.00	1.00	1.00	1.00	1.00	FALSE	2
g_planctomycete	1.00	1.00	1.00	1.00	1.60	1.00	1.00	1.00	1.00	1.00	FALSE	0
g_Planomicrobium	1.00	1.00	1.00	1.00	1.33	1.00	1.00	1.00	1.00	1.00	FALSE	0
g_Polaribacter	1.00	1.00	1.00	1.00	1.00	1.00	1.00	1.00	1.00	2.28	FALSE	0
g_Prevotella	1.00	1.00	1.00	1.02	1.11	1.00	1.00	1.00	1.00	1.00	FALSE	1
g_Proteiniclasticum	1.00	1.00	1.00	1.00	2.12	1.00	1.00	1.00	1.00	1.00	FALSE	0
g_Pseudidiomarina	1.00	1.00	1.00	1.00	1.98	1.00	1.00	1.00	1.00	1.00	FALSE	4
g_Pseudoalteromonas	1.00	1.00	1.00	1.00	1.15	1.00	1.00	1.00	2.27	2.48	FALSE	0
g_Pseudomonas	1.00	1.00	1.00	1.44	2.79	1.56	2.67	4.07	4.58	5.69	FALSE	8
g_Psychrobacter	1.00	1.00	1.00	1.05	1.30	1.72	2.50	3.46	3.88	7.75	TRUE	450

g_Psychroflexus	1.00	1.00	1.00	1.00	1.13	1.00	1.00	1.00	1.00	1.00	FALSE	1
g_Psychroserpens	1.00	1.00	1.00	1.00	1.29	1.00	1.00	1.00	1.00	1.00	FALSE	0
g_Ralstonia	1.00	1.00	1.00	1.06	2.28	1.00	1.00	1.00	1.26	1.78	FALSE	0
g_Rhodopirellula	1.00	1.00	1.00	1.02	1.12	1.00	1.00	1.00	1.00	1.00	FALSE	1
g_Rhodovulum	1.00	1.00	1.00	1.00	1.27	1.00	1.00	1.00	1.00	1.00	FALSE	0
g_Roseivirga	1.00	1.09	1.22	1.74	2.42	1.00	1.00	1.00	1.00	1.00	FALSE	1
g_Ruminococcus	1.00	1.00	1.00	1.00	1.20	1.00	1.00	1.00	1.00	1.17	FALSE	2
g_Ruminococcus	1.00	1.54	2.11	2.58	5.15	1.10	1.30	2.00	2.93	2.94	FALSE	1
g_Salegentibacter	1.00	1.00	1.87	3.77	29.44	4.25	4.45	5.12	6.75	47.03	FALSE	1
g_Salinibacterium	1.00	1.00	1.00	1.00	1.14	1.00	1.00	1.00	1.00	1.34	FALSE	0
g_Shewanella	1.00	1.00	1.00	1.02	1.70	1.00	1.00	1.00	1.00	1.00	FALSE	0
g_Sphingomonas	1.00	1.00	1.00	1.00	1.46	1.00	1.00	1.00	1.00	1.00	FALSE	0
g_Sphingopyxis	1.00	1.00	1.00	1.00	1.08	1.00	1.00	1.00	1.00	1.00	FALSE	0
g_Staphylococcus	1.00	1.00	1.00	1.00	1.12	1.00	1.00	1.00	1.00	1.00	FALSE	0
g_Stenotrophomonas	1.00	1.00	1.00	1.00	1.22	1.00	1.00	1.00	1.00	1.00	FALSE	0
g_Sutterella	1.00	1.00	1.00	1.00	1.22	1.00	1.00	1.00	1.00	1.00	FALSE	0
g_Thalassospira	1.00	1.00	1.10	1.31	4.03	1.00	1.00	1.00	1.00	1.00	FALSE	0
g_Thermus	1.00	1.00	1.00	1.00	1.26	1.00	1.00	1.00	1.00	1.00	FALSE	0
g_Thiomicrospira	1.00	1.00	1.00	1.04	3.91	1.00	1.00	1.00	1.00	1.00	FALSE	0
g_Ulvibacter	1.00	1.00	1.00	1.00	3.16	1.00	1.00	1.00	1.00	1.00	FALSE	0
g_Winogradskyella	1.00	1.00	1.00	1.15	3.07	1.00	1.00	1.00	1.00	1.00	FALSE	1

Table S5 Imputed metabolic functions of bacterial community. Values are percentage.

Functional groups	All sediments	Inshore sediment									Offshore sediment					
		JB01	R02	R04	R08	R09	R14	R18	R19	mean	JB02	JB03	JB04	JB05	R17	mean
Chemoheterotrophy	79.05	75.76	47.68	86.91	28.59	82.35	74.24	62.64	80.17	67.29	97.59	96.11	98.63	96.78	99.77	97.77
Aerobic chemoheterotrophy	65.82	60.63	35.84	12.34	22.88	79.71	50.17	59.90	73.45	49.37	87.13	88.90	96.78	88.20	99.17	92.04
Fermentation	14.62	14.96	14.65	81.97	10.16	2.77	22.53	2.91	7.46	19.68	12.85	7.73	2.00	9.37	0.65	6.52
Nitrate reduction	2.63	1.14	1.03	10.42	1.07	0.43	0.95	0.44	12.86	3.54	3.08	1.10	0.26	1.30	0.11	1.17
Dissimilatory nitrate reduction to ammonium	1.25	0.17	0.65	8.16	0.99	0.23	0.30	0.14	0.87	1.44	2.79	0.73	0.20	0.97	0.07	0.95
Denitrification	0.05	0.00	0.00	0.00	0.01	0.08	0.02	0.25	0.07	0.05	0.01	0.16	0.00	0.00	0.02	0.04
Respiration of sulfur compounds	0.13	0.83	0.15	0.00	0.40	0.15	0.00	0.06	0.06	0.21	0.00	0.01	0.02	0.02	0.00	0.01
Fumarate respiration	1.24	0.16	0.64	8.13	0.97	0.22	0.30	0.14	0.86	1.43	2.76	0.67	0.19	0.96	0.07	0.93
Arsenate respiration	0.01	0.01	0.00	0.02	0.01	0.00	0.00	0.00	0.00	0.01	0.01	0.03	0.00	0.01	0.00	0.01
Xylanolysis	1.91	2.33	1.62	10.73	0.97	0.28	3.50	0.27	1.00	2.59	1.62	1.02	0.28	1.08	0.12	0.82
Cellulolysis	0.04	0.07	0.10	0.00	0.23	0.05	0.00	0.00	0.00	0.06	0.00	0.00	0.00	0.01	0.00	0.00
Chitinolysis	0.02	0.00	0.00	0.14	0.00	0.00	0.00	0.00	0.01	0.02	0.04	0.02	0.01	0.01	0.00	0.02
Hydrocarbon degradation	1.72	2.94	1.17	1.77	0.21	1.81	2.39	1.41	2.65	1.79	2.51	1.63	1.94	0.34	1.53	1.59
Aromatic compound degradation	0.39	0.09	0.16	2.12	0.29	0.06	0.13	0.06	0.36	0.41	0.90	0.34	0.10	0.43	0.03	0.36
Aromatic hydrocarbon degradation	0.29	0.07	0.13	1.64	0.16	0.05	0.09	0.03	0.27	0.31	0.67	0.24	0.08	0.32	0.02	0.27
Plastic degradation	0.15	0.02	0.07	0.83	0.10	0.03	0.05	0.02	0.14	0.16	0.33	0.09	0.05	0.17	0.01	0.13
Methylotrophy	0.24	0.23	0.90	0.02	0.01	0.00	1.96	0.00	0.00	0.39	0.00	0.00	0.00	0.00	0.00	0.00
Dark sulfur oxidation	1.75	4.82	0.59	0.19	6.12	2.77	1.44	0.69	3.00	2.45	0.36	2.47	0.00	0.01	0.28	0.62
Manganese oxidation	0.06	0.16	0.00	0.01	0.01	0.43	0.01	0.00	0.00	0.08	0.04	0.08	0.01	0.00	0.00	0.03
Chloroplasts	0.13	0.04	0.82	0.01	0.00	0.36	0.01	0.19	0.01	0.18	0.00	0.00	0.12	0.11	0.00	0.05
Photoheterotrophy	0.04	0.00	0.00	0.00	0.02	0.00	0.00	0.47	0.08	0.07	0.00	0.00	0.00	0.00	0.00	0.00

Table S6 Taxonomic statistic of 629 isolates obtained by culture-dependent method.

Stations	Total	JB01	JB02	JB03	JB04	JB05	R02	R04	R08	R09	R14	R17	R18	R19
Proteobacteria	463	16	36	19	31	30	39	29	41	48	32	38	51	53
Caulobacterales	2	0	0	0	0	0	0	0	0	2	0	0	0	0
Kiloniellales	17	1	6	4	0	0	1	0	1	1	0	2	1	0
Rhizobiales	46	1	4	1	2	6	1	4	7	8	2	4	6	0
Rhodobacterales	102	1	11	0	16	2	12	7	7	6	6	12	8	14
Sphingomonadales	33	1	5	3	1	2	2	3	2	3	0	3	3	5
Alteromonadales	106	3	0	8	4	14	7	6	10	21	7	13	8	5
Oceanospirillales	89	7	8	2	5	4	2	4	6	4	13	3	20	11
Pseudomonadales	60	2	0	0	3	0	14	5	7	1	4	1	5	18
Thiotrichales	6	0	0	1	0	2	0	0	1	2	0	0	0	0
Vibrionales	2	0	2	0	0	0	0	0	0	0	0	0	0	0
Bacteroidetes	112	7	12	10	5	16	2	10	6	9	10	7	14	4
Cytophagales	41	1	3	3	3	11	0	2	1	2	5	0	8	2
Flavobacteriales	71	6	9	7	2	5	2	8	5	7	5	7	6	2
Actinobacteria	32	0	6	1	1	2	2	3	0	1	3	2	11	0
Actinomycetales	32	0	6	1	1	2	2	3	0	1	3	2	11	0
Firmicutes	22	2	2	0	1	1	1	1	2	0	4	1	7	0
Bacillales	22	2	2	0	1	1	1	1	2	0	4	1	7	0
Total isolates	629	25	56	30	38	49	44	43	49	58	49	48	83	57

Table S7 13 OTUs only detected by culture-dependent technique.

Representative isolate	Number of isolates	Taxonomy
R14-L2	72	k__Bacteria; p__Proteobacteria; c__Alphaproteobacteria; o__Rhodobacterales; f__Rhodobacteraceae; g__
R02-M33	15	k__Bacteria; p__Firmicutes; c__Bacilli; o__Bacillales; f__Bacillaceae; g__Bacillus
JB01-H21	13	k__Bacteria; p__Proteobacteria; c__Gammaproteobacteria; o__Pseudomonadales; f__Pseudomonadaceae; g__Pseudomonas
R09-M3	8	k__Bacteria; p__Proteobacteria; c__Alphaproteobacteria; o__Rhizobiales; f__Aurantimonadaceae; g__Martelella
R14-M11	3	k__Bacteria; p__Actinobacteria; c__Actinobacteria; o__Actinomycetales; f__Microbacteriaceae; g__Microbacterium
JB03-H2	2	k__Bacteria; p__Actinobacteria; c__Actinobacteria; o__Actinomycetales; f__Nocardioidaceae; g__
JB03-H15	2	k__Bacteria; p__Bacteroidetes; c__Cytophagia; o__Cytophagales; f__Flammeovirgaceae; g__
JB04-L15	2	k__Bacteria; p__Actinobacteria; c__Actinobacteria; o__Actinomycetales; f__Nocardiaceae; g__Rhodococcus
JB05-M4	2	k__Bacteria; p__Proteobacteria; c__Alphaproteobacteria; o__Rhodobacterales; f__Rhodobacteraceae; g__
JB05-H15	1	k__Bacteria; p__Proteobacteria; c__Alphaproteobacteria; o__Rhizobiales; f__Hyphomicrobiaceae; g__Devosia
R17-H7	1	k__Bacteria; p__Actinobacteria; c__Actinobacteria; o__Actinomycetales; f__Microbacteriaceae; g__Agrococcus
R17-H8A	1	k__Bacteria; p__Actinobacteria; c__Actinobacteria; o__Actinomycetales; f__Micrococcaceae; g__
R17-H24	1	k__Bacteria; p__Proteobacteria; c__Gammaproteobacteria; o__Alteromonadales; f__Alteromonadaceae; g__Marinobacter

Table S8 Top 10 OTUs preferred by culture-dependent technique. Abundance ratio was calculated by dividing the abundance of culture-dependent OTUs with the abundance of culture-independent OTUs.

OTU	Abundance ratio	Taxonomy
OTU121	106.90	k__Bacteria; p__Actinobacteria; c__Actinobacteria; o__Actinomycetales; f__Microbacteriaceae; g__Salinibacterium
OTU84	73.57	k__Bacteria; p__Proteobacteria; c__Alphaproteobacteria; o__Rhizobiales; f__Aurantimonadaceae; g__
OTU104	47.52	k__Bacteria; p__Proteobacteria; c__Gammaproteobacteria; o__Pseudomonadales; f__Pseudomonadaceae; g__Pseudomonas
OTU86	43.15	k__Bacteria; p__Proteobacteria; c__Alphaproteobacteria; o__Caulobacterales; f__Caulobacteraceae; g__Brevundimonas
OTU113	43.15	k__Bacteria; p__Bacteroidetes; c__Flavobacteriia; o__Flavobacteriales; f__Flavobacteriaceae; g__Zhouia
OTU166	32.36	k__Bacteria; p__Proteobacteria; c__Deltaproteobacteria; o__DTB120; f__; g__
OTU304	26.81	k__Bacteria; p__Proteobacteria; c__Gammaproteobacteria; o__Pseudomonadales; f__Pseudomonadaceae; g__
OTU188	21.57	k__Bacteria; p__Proteobacteria; c__Alphaproteobacteria; o__Rhizobiales; f__Hyphomicrobiaceae; g__Devosia
OTU67	14.69	k__Bacteria; p__Bacteroidetes; c__Cytophagia; o__Cytophagales; f__Cyclobacteriaceae; g__
OTU68	14.43	k__Bacteria; p__Firmicutes; c__Bacilli; o__Bacillales; f__Planococcaceae; g__Planomicrobium
OTU368	12.24	k__Bacteria; p__Proteobacteria; c__Gammaproteobacteria; o__Alteromonadales; f__Alteromonadaceae; g__Glaciecola
OTU244	12.13	k__Bacteria; p__Proteobacteria; c__Alphaproteobacteria; o__Kiloniellales; f__Kiloniellaceae; g__Thalassospira
OTU29	12.06	k__Bacteria; p__Proteobacteria; c__Gammaproteobacteria; o__Oceanospirillales; f__Halomonadaceae; g__Halomonas
OTU83	11.92	k__Bacteria; p__Actinobacteria; c__Actinobacteria; o__Actinomycetales; f__Nocardioidaceae; g__Aeromicrobium
OTU132	11.62	k__Bacteria; p__Firmicutes; c__Bacilli; o__Bacillales; f__Bacillaceae; g__Marinibacillus; g__
OTU6	10.18	k__Bacteria; p__Bacteroidetes; c__Cytophagia; o__Cytophagales; f__Cyclobacteriaceae; g__

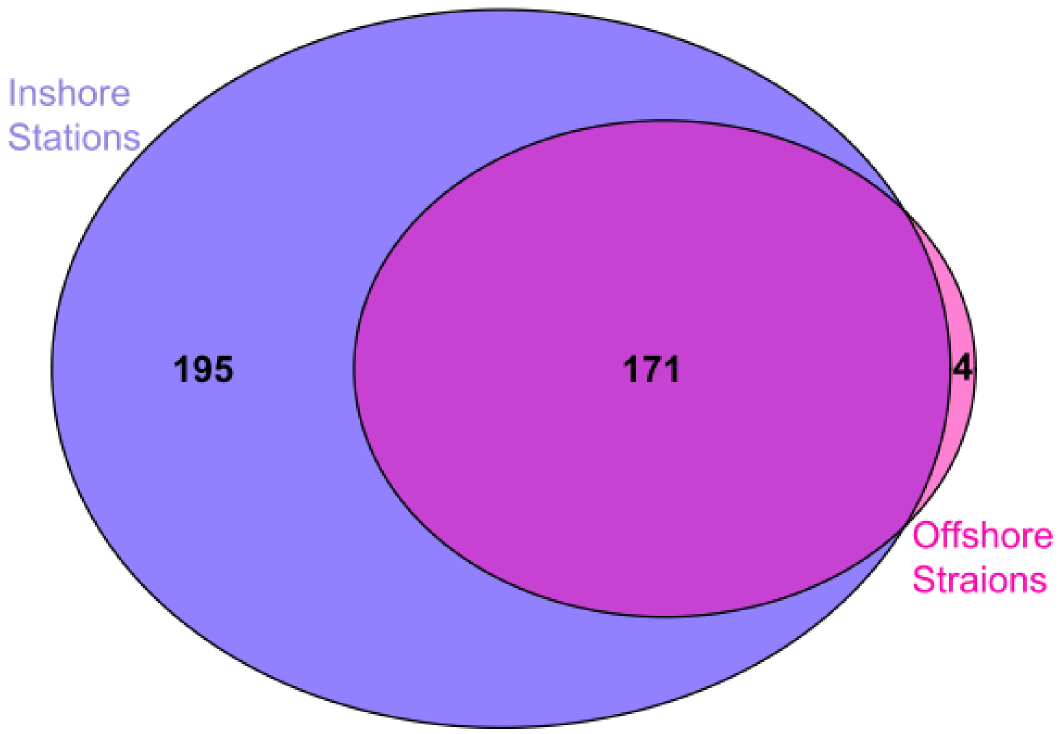

Fig. S1 Venn diagram of OTUs detected in inshore (violet) and offshore samples (pink).

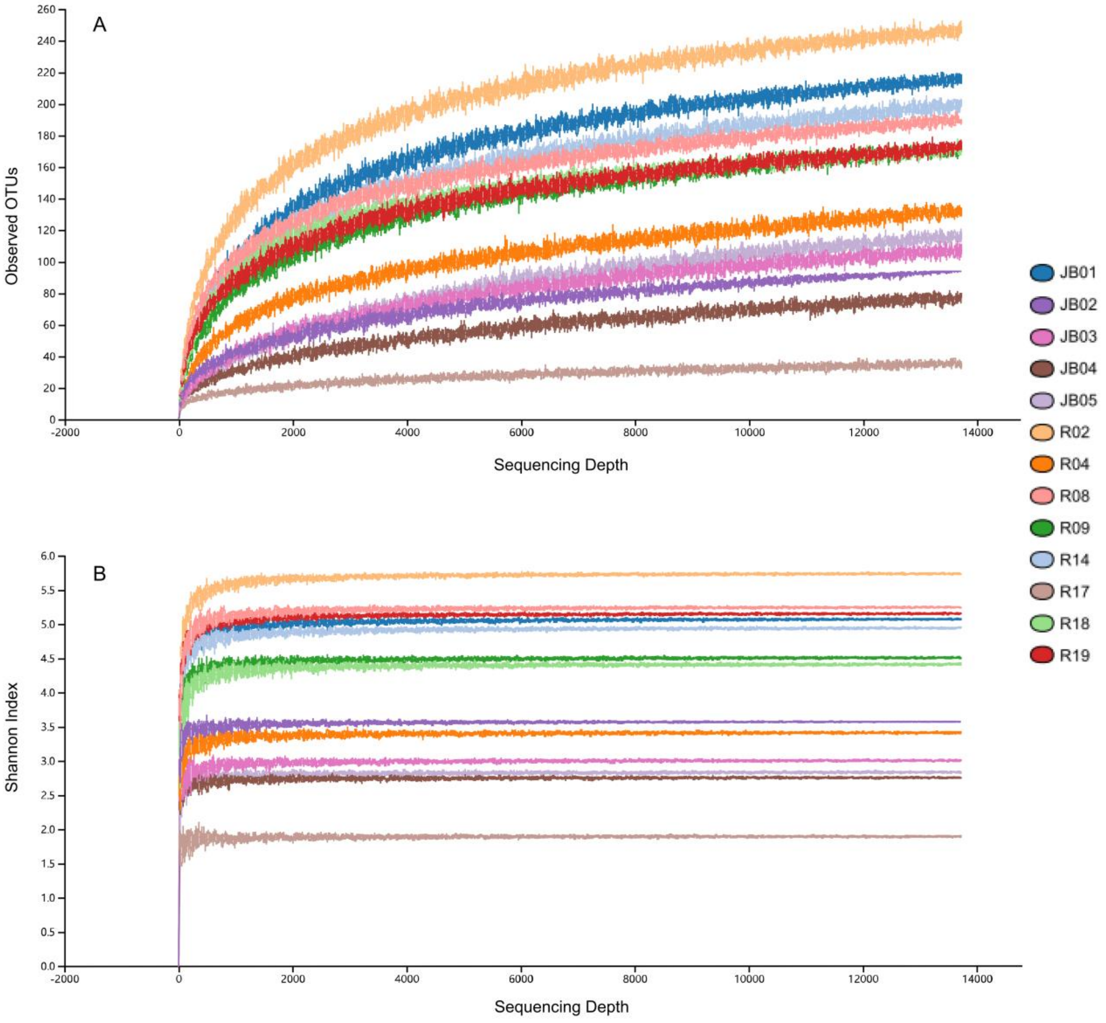

Fig. S2 Rarefaction curves of high-throughput sequencing of 16S rRNA gene amplicons. A, rarefaction curves based on observed OTUs; B, rarefaction curves based on Shannon index.

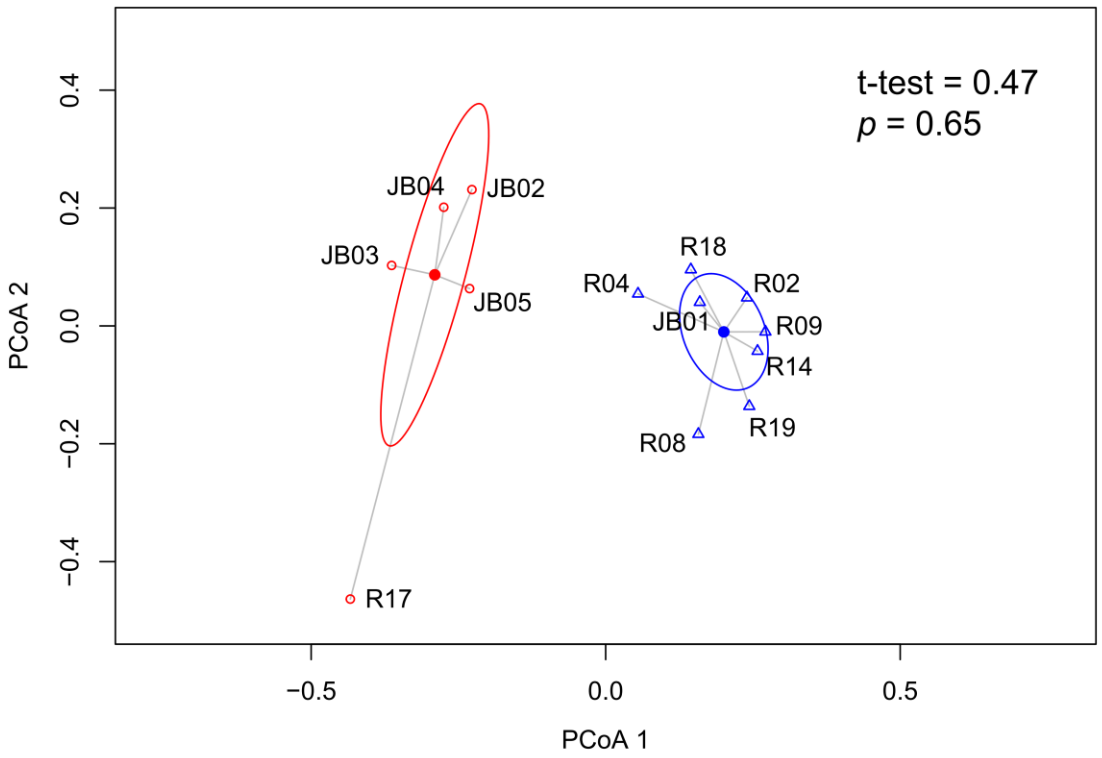

Fig. S3 Distribution of Jaccard dissimilarity within inshore group and offshore group, respectively. Jaccard distances between samples and group centroids were handled by reducing the original distances to principal coordinates. Solid circles were group centroids; red, offshore group; blue, inshore group.

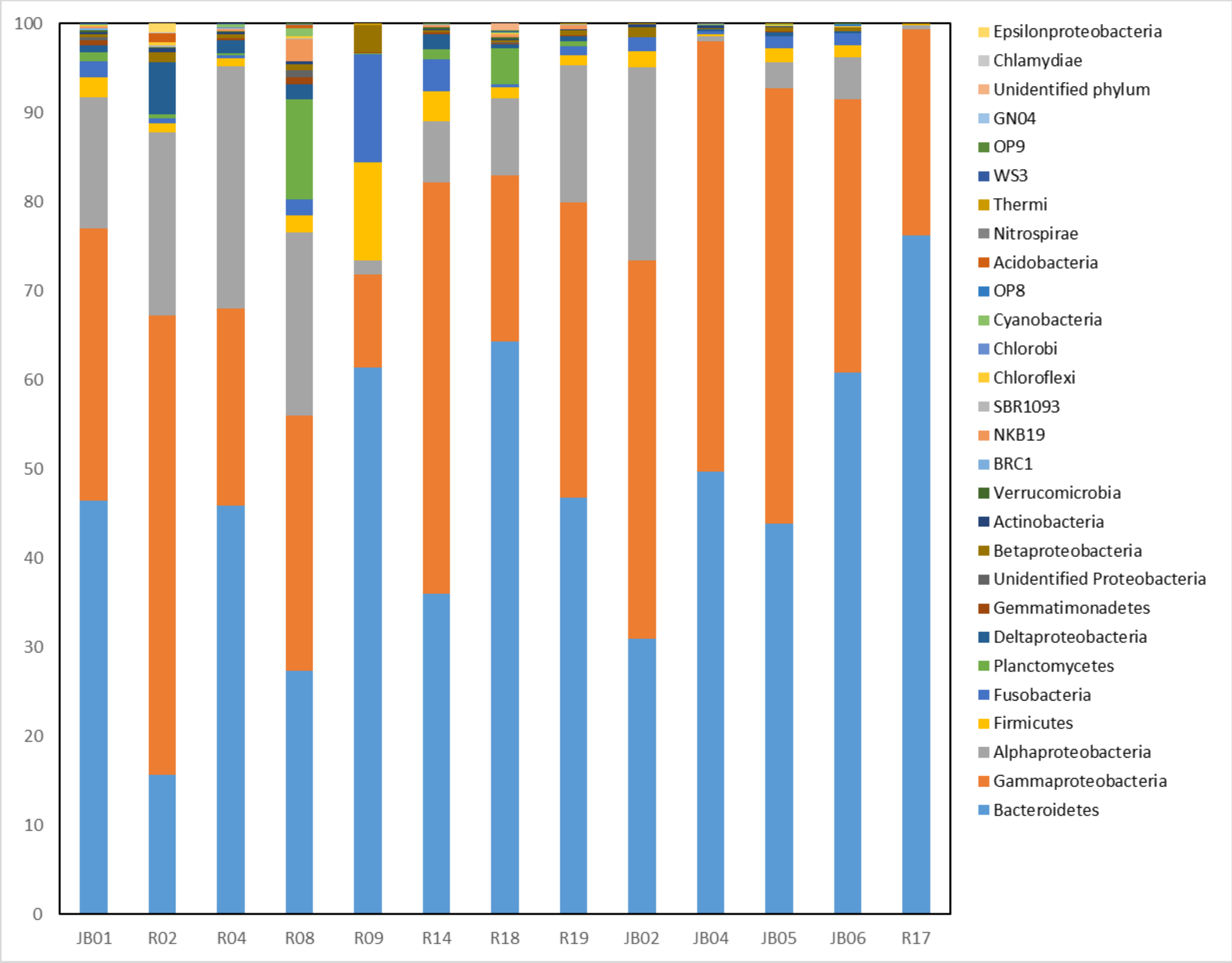

Fig. S4 Composition of bacterial community in the Ross Sea sediments. Bar charts showed the relative abundance of bacterial taxa in each of the sediments at phylum level (*Proteobacteria* was presented at class level).

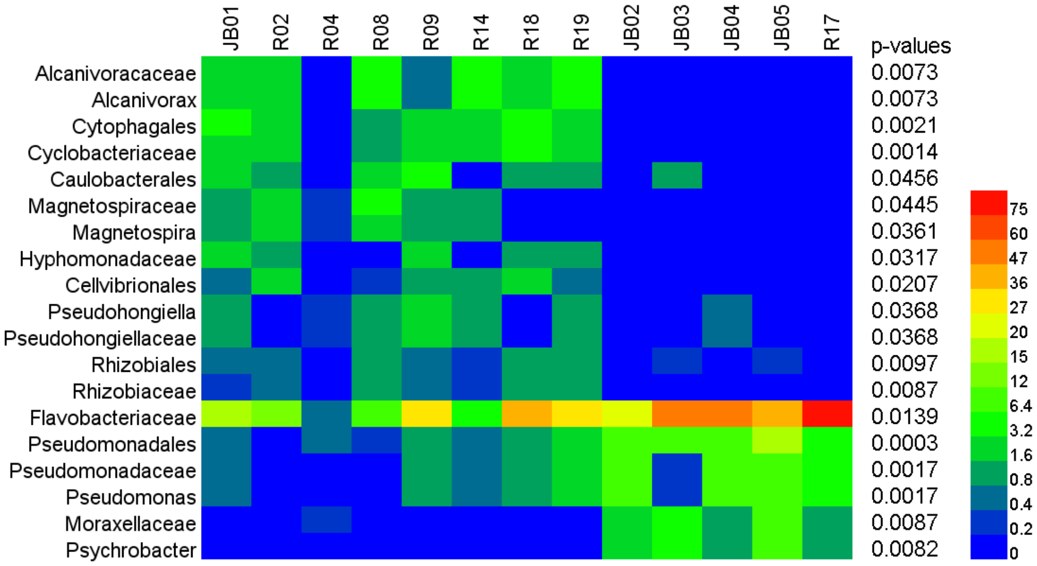

Fig. S5 Indicative taxa with significantly different distributions between inshore and offshore group. Welch's t-test was performed by R. Color indicates the abundance (%) of given taxa.

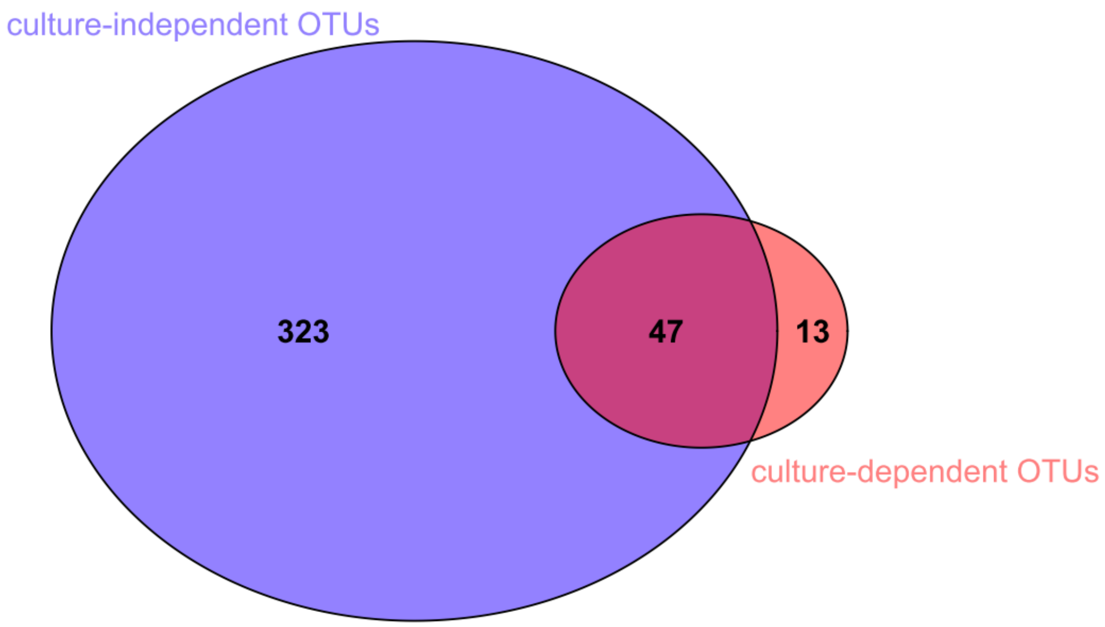

Fig. S6 Venn diagram of OTUs revealed by culture-dependent (coral) and -independent (violet) approaches.

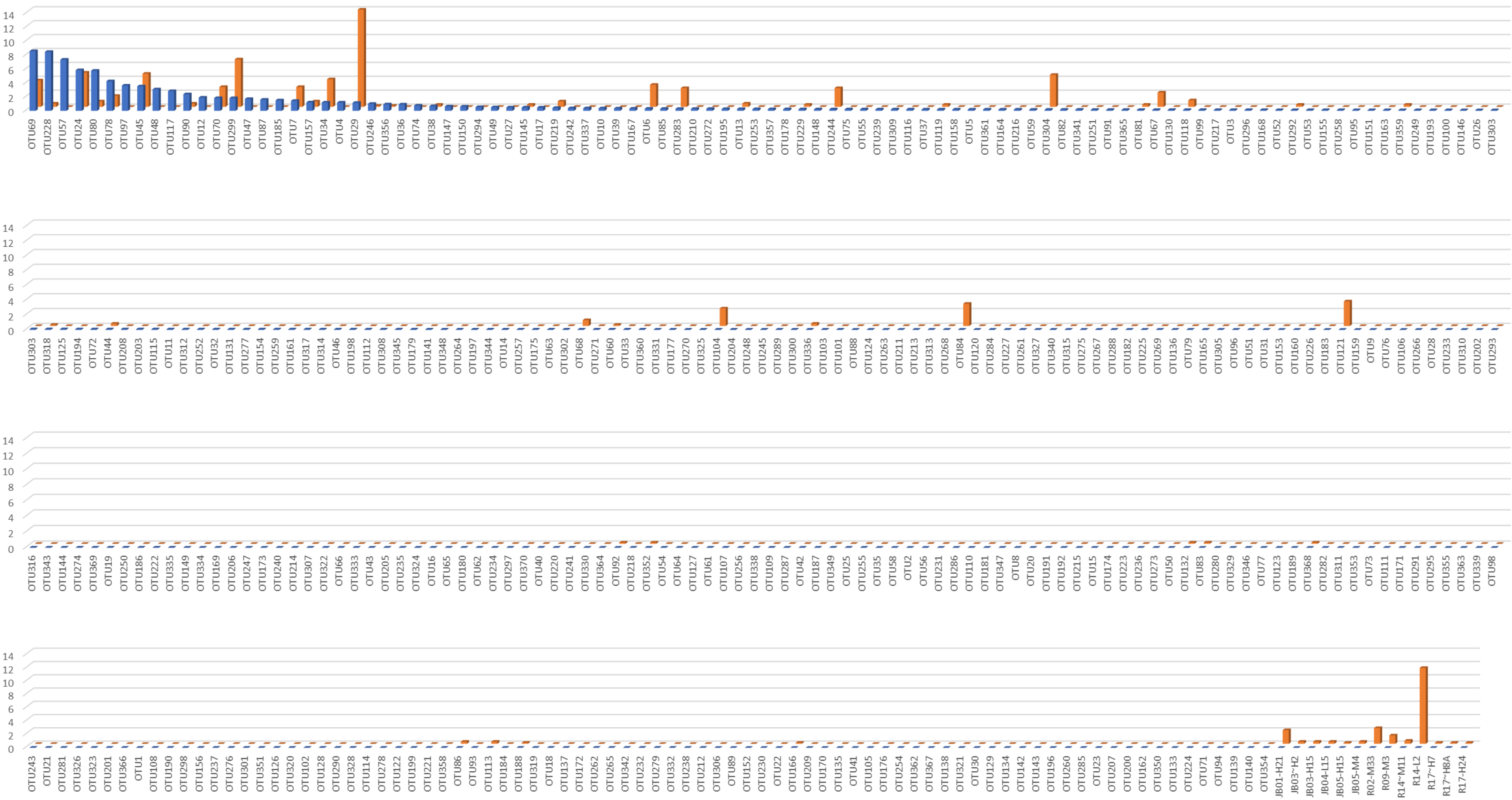

Fig. S7 Comparison of OTU abundance revealed by culture-dependent and -independent approaches. The Y-axis showed the percentage of a given OTU.
